# Reductive dechlorination of 1,2-dichloroethane in the presence of chloroethenes and 1,2-dichloropropane as co-contaminants

**DOI:** 10.1007/s00253-019-09985-8

**Published:** 2019-06-28

**Authors:** Peng Peng, Uwe Schneidewind, Pieter Jan Haest, Tom N. P. Bosma, Anthony S. Danko, Hauke Smidt, Siavash Atashgahi

**Affiliations:** 1 Laboratory of Microbiology, Wageningen University & Research, Wageningen, The Netherlands; 2 Department of Civil and Environmental Engineering, Western University, London, ON Canada; 3 Advanced Groundwater Techniques (AGT), Aartselaar, Belgium; 4 Division of Geology, Department of Earth and Environmental Sciences, KU Leuven, Heverlee, Belgium; 5 Centre for Natural Resources and the Environment (CERENA), Department of Mining Engineering, University of Porto (FEUP), Porto, Portugal

**Keywords:** 1,2-Dichloroethane, Co-contaminants, *Dehalococcoides*, *Dehalogenimonas*, Dechlorination kinetics

## Abstract

1,2-Dichloroethane (1,2-DCA) is one of the most abundant manmade chlorinated organic contaminants in the world. Reductive dechlorination of 1,2-DCA by organohalide-respiring bacteria (OHRB) can be impacted by other chlorinated contaminants such as chloroethenes and chloropropanes that can co-exist with 1,2-DCA at contaminated sites. The aim of this study was to evaluate the effect of chloroethenes and 1,2-dichloropropane (1,2-DCP) on 1,2-DCA dechlorination using sediment cultures enriched with 1,2-DCA as the sole chlorinated compound (EA culture) or with 1,2-DCA and tetrachloroethene (PCE) (EB culture), and to model dechlorination kinetics. Both cultures contained *Dehalococcoides* as most predominated OHRB, and *Dehalogenimonas* and *Geobacter* as other known OHRB. In sediment-free enrichments obtained from the EA and EB cultures, dechlorination of 1,2-DCA was inhibited in the presence of the same concentrations of either PCE, vinyl chloride (VC), or 1,2-DCP; however, concurrent dechlorination of dual chlorinated compounds was achieved. In contrast, 1,2-DCA dechlorination completely ceased in the presence of *cis*-dichloroethene (cDCE) and only occurred after cDCE was fully dechlorinated. In turn, 1,2-DCA did not affect dechlorination of PCE, cDCE, VC, and 1,2-DCP. In sediment-free enrichments obtained from the EA culture, *Dehalogenimonas* 16S rRNA gene copy numbers decreased 1–3 orders of magnitude likely due to an inhibitory effect of chloroethenes. Dechlorination with and without competitive inhibition fit Michaelis-Menten kinetics and confirmed the inhibitory effect of chloroethenes and 1,2-DCP on 1,2-DCA dechlorination. This study reinforces that the type of chlorinated substrate drives the selection of specific OHRB, and indicates that removal of chloroethenes and in particular cDCE might be necessary before effective removal of 1,2-DCA at sites contaminated with mixed chlorinated solvents.

**Electronic supplementary material:**

The online version of this article (10.1007/s00253-019-09985-8) contains supplementary material, which is available to authorized users.

## Introduction

Understanding biodegradation bottlenecks has been a major objective in efforts to harness the metabolic potential of microorganisms for bioremediation of sites contaminated with organic pollutants (Atashgahi et al. 2018; Meckenstock et al. 2015; Vandermaesen et al. 2016). An important class of such contaminants comprises chlorinated solvents such as chloroethenes, chloroethanes, and chloropropanes that have adverse effects on human and environmental health (EPA 2018; Weatherill et al. 2018). Organohalide respiration (OHR) is an example of a microbial metabolism that has been successfully harnessed for engineered remediation of sites contaminated with chlorinated solvents (Atashgahi et al. 2017; Edwards 2014; Ellis et al. 2000). This process is mediated by organohalide-respiring bacteria (OHRB) belonging to distinct genera within the phyla *Chloroflexi* (e.g., *Dehalococcoides* and *Dehalogenimonas*), *Firmicutes* (e.g., *Dehalobacter* and *Desulfitobacterium*), and *Proteobacteria* (e.g., *Sulfurospirillum* and *Geobacter*) (Atashgahi et al. 2016).

One of the challenges of bioremediation is the presence of mixtures of organohalogens at contaminated sites. During dechlorination of co-mingled organohalogens, bioattenuation of specific chlorinated solvents has been shown to be prone to inhibition due to the inhibitory effect of dechlorination intermediates on OHRB, their reductive dehalogenase enzymes, and their syntrophic partners (Chan et al. 2011; Dillehay et al. 2014; Grostern et al. 2009; Mayer-Blackwell et al. 2016). For instance, 1,1,1-trichloroethane (1,1,1-TCA) was shown to strongly inhibit chloroethene-reductive dehalogenases of *Dehalococcoides* (Chan et al. 2011). Moreover, long-term exposure to 1,2-dichloroethane (1,2-DCA) was shown to shift the *Dehalococcoides* community within a microbial consortium from vinyl chloride (VC) reductive dehalogenase gene (*vcrA*)-containing *Dehalococcoides* to trichloroethene (TCE) reductive dehalogenase gene (*tceA*)-containing *Dehalococcoides*, leading to diminished VC transforming ability (Mayer-Blackwell et al. 2016). In turn, kinetic modeling using the same culture revealed that 1,2-DCA dechlorination was strongly inhibited by *cis*-dichloroethene (cDCE), and efficient 1,2-DCA dechlorination occurred only when cDCE was completely dechlorinated to VC (Mayer-Blackwell et al. 2016). In another study, the presence of 1,1,2-trichloroethane (1,1,2-TCA) and 1,2-dichloropropane (1,2-DCP) inhibited 1,2-DCA dechlorination by *Dehalogenimonas lykanthroporepellens* BL-DC-9 and *D. alkenigignens* IP3-3 (Dillehay et al. 2014). An improved understanding of such inhibitory effects can aid in designing bioremediation approaches for sites contaminated with a mixture of chloroethenes, chloroethanes, and/or chloropropanes (Dillehay et al. 2014; Field and Sierra-Alvarez 2004; Mayer-Blackwell et al. 2016).

Different modeling approaches of varying complexity have been developed to understand the reductive dechlorination of chloroethenes with and without competitive inhibition (Chambon et al. 2013). The description of the reaction kinetics varies from first-order (Corapcioglu et al. 2004; Da Silva and Alvarez 2008) to the more elaborate Michaelis-Menten equations (Garant and Lynd 1998; Haston and McCarty 1999) or Monod kinetics if the responsible OHRB can be sufficiently quantified (Yu and Semprini 2004). The latter two kinetic modeling approaches have been applied at lab and field scales to study competitive inhibition (Christ and Abriola 2007; Lai and Becker 2013; Yu et al. 2005), self-inhibition (Haest et al. 2010a), electron donor limitation (Cupples et al. 2004), dechlorination in the presence of multiple bacterial species (Brovelli et al. 2012; Duhamel and Edwards 2006; Haest et al. 2010b), and dechlorination in conjunction with fermentation, sulfate reduction, or methanogenesis (Kouznetsova et al. 2010; Malaguerra et al. 2011). The Michaelis-Menten and Monod kinetic modeling approaches can also be used to study the dechlorination of chloroethanes or chloropropanes; however, examples in literature are rare. Notable exceptions are Mayer-Blackwell et al. (2016) who studied concurrent dechlorination of 1,2-DCA and cDCE, and Colombani et al. (2014) who studied 1,2-DCA degradation under the influence of salt water intrusion.

The aim of this study was to evaluate the impact of chloroethenes and 1,2-DCP on 1,2-DCA dechlorination using enrichment cultures containing *Dehalococcoides*, *Geobacter*, and *Dehalogenimonas* as the known OHRB. The prime focus was to obtain an improved understanding of 1,2-DCA dechlorination, which is the most abundant chlorinated organic contaminant worldwide (Field and Sierra-Alvarez 2004). 1,2-DCA can be dihaloeliminated to ethene by diverse OHRB including members of the genera *Dehalococcoides* (Maymó-Gatell et al. 1999; Parthasarathy et al. 2015; Wang and He 2013), *Geobacter* (Duhamel and Edwards 2006), *Dehalogenimonas* (Maness et al. 2012), *Desulfitobacterium* (Low et al. 2019; Marzorati et al. 2007), and *Dehalobacter* (Grostern and Edwards 2009). 1,2-DCA has been found to co-exist with chloroethenes and/or chloropropanes at many contaminated sites (Dillehay et al. 2014; Mayer-Blackwell et al. 2016). Despite some reports on suppression of 1,2-DCA dechlorination by co-occurring chloroethenes, chloroethanes, and bromoethanes (Dillehay et al. 2014; Mayer-Blackwell et al. 2016; Yu et al. 2013), comprehensive studies on the interaction between 1,2-DCA, 1,2-DCP, and chloroethenes with respect to their dechlorination in complex organohalide-respiring microbial consortia are limited.

In this study, in cultures amended with one or two chlorinated compounds (1,2-DCA with either tetrachloroethene (PCE), cDCE, VC, or 1,2-DCP), the impact of co-contaminants on OHRB was investigated by quantifying 16S ribosomal RNA (rRNA) genes of known OHRB. The dechlorination reactions were approximated with Michaelis-Menten kinetics taking into account competitive inhibition. Parameter estimation was performed using AMALGAM (Vrugt and Robinson 2007), a multi-objective, multi-method (ensemble) evolutionary optimization procedure to account for the high correlation among the parameters describing dechlorination kinetics and the existence of multiple solutions. Results showed that all applied chlorinated compounds inhibited 1,2-DCA dechlorination, whereas 1,2-DCA had no pronounced inhibitory effect on the dechlorination of other chlorinated compounds.

## Materials and methods

### Chemicals

1,2-DCA, chloroethenes, 1,2-DCP, ethene, and propene were purchased from Sigma-Aldrich, and were used directly in the following experiments. Lactate stock solution (1 M) was prepared from 60% sodium dl-lactate solution (Sigma-Aldrich). Other organic and inorganic chemicals were of analytical grade and were used without further purification.

### Sediment collection and enrichment set-up

Surface sediment samples (down to 15 cm below surface) were collected from a wetland in Estarreja, Portugal. This site has a long history of contamination with agrochemical and fine chemistry effluents (Carvalho et al. 2005). Sediment enrichment cultures were set up in 120-mL serum bottles using 10 g of wet sediment and 50 mL of an anoxic medium as described previously (Stams et al. 1993). Resazurin (0.005 g/L) and Na_2_S·9H_2_O (0.48 g/L) were added as redox indicator and reducing reagent, respectively. The headspace of the bottles was exchanged with N_2_ and CO_2_ (80:20%, 140 kPa). Lactate (3 mM) was used as the carbon source and electron donor. The electron acceptors for the sediment cultures were PCE (10 μmol/bottle, designated EA culture) and PCE plus 1,2-DCA (10 μmol/bottle each, designated EB culture). The bottles were sealed with Teflon lined butyl rubber stoppers and aluminum crimp caps (GRACE, MD, USA) and incubated statically in the dark at 20 °C. The EA and EB sediment cultures were spiked 21 times with their respective chlorinated electron acceptors during enrichment. After dechlorination of each spike of the chlorinated substrates to ethene, the headspace of the cultures was flushed with N_2_ and CO_2_ (80:20%) for three times (3 min for each run and 10 min rest in between each flushing cycle) before re-amendment of the respective chlorinated substrate(s). In the last three spikes, the concentration of each chlorinated substrate(s) was increased stepwise from 10 μmol/bottle to 25 and 40 μmol/bottle. To avoid toxicity of the chlorinated compounds in the following experiments, each single chlorinated substrate was added at 25 μmol/bottle unless otherwise stated.

**Fig. 1:**
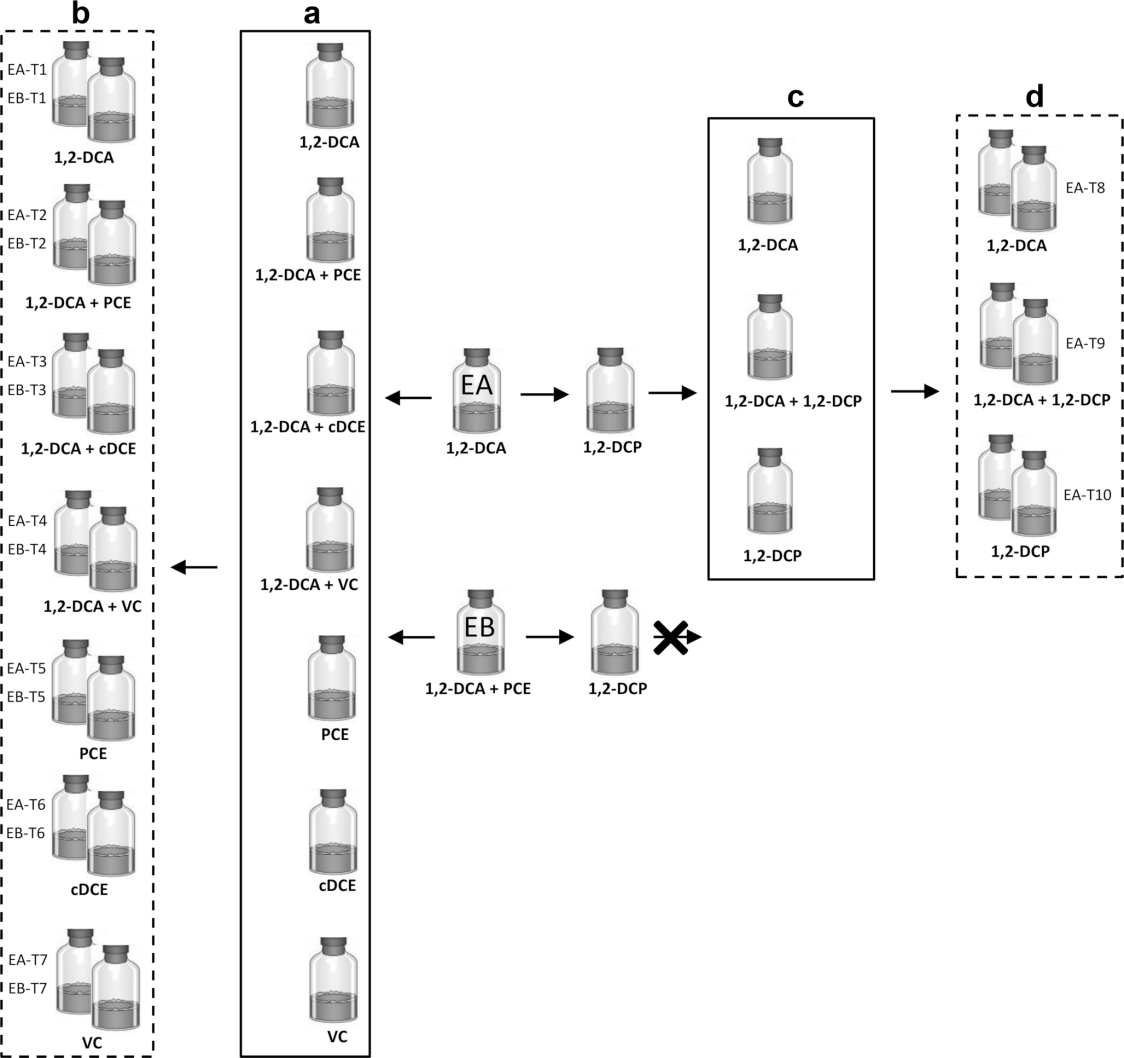
Schematic representation of the experimental set-up. Cultures in box **a** received inoculum from either EA or EB culture. Cultures in dashed boxes (**b**, **d**) were used for the kinetic study. The EB culture was not able to dechlorinate 1,2-DCP and hence not used for the kinetic study performed using cultures in box **d**

Sediment-free cultures were obtained by transferring the EA and EB sediment cultures as the following: a 5% slurry from the EA and EB sediment cultures was transferred into bottles containing fresh anoxic medium with lactate (5 mM) and single or double chlorinated substrates, i.e., 1,2-DCA either alone or with PCE, cDCE, or VC (Fig. 1(a)). Each transfer culture was amended with three spikes of the respective chlorinated substrate(s), and then diluted to duplicate cultures (50% inoculum, Fig. 1(b)) and amended with another three spikes of the respective chlorinated substrate(s). In parallel, a 5% slurry from the EA and EB sediment cultures was also transferred into bottles containing 1,2-DCP (10 μmol/bottle). Only the EA transfer culture showed 1,2-DCP dechlorination. Therefore, only this culture was subsequently transferred (5% inoculum) to fresh anoxic medium with either 1,2-DCP, 1,2-DCA, or a mixture of 1,2-DCP and 1,2-DCA (Fig. 1(c)). After depletion of three spikes of the respective chlorinated substrate, these cultures were diluted to duplicate cultures (50% inoculum, Fig. 1(d)) and amended with another three spikes of the respective chlorinated substrate(s).

The dechlorination pattern of the last (third) spike of chlorinated substrates(s) (Fig. 1(b, d)) was used for kinetic modeling. To study the relief of inhibition by different chlorinated compounds on 1,2-DCA dechlorination, the cultures amended with double chlorinated substrates were subsequently spiked only with 1,2-DCA.

### DNA extraction and quantitative PCR

After dechlorination of each spike of the chlorinated compounds during the last three spikes (amended respectively with 10, 25, and 40 μmol/bottle each compound) in the EA and EB sediment cultures (Fig. 1), 2 mL slurry samples were taken for DNA extraction. After dechlorination of the third spike of chlorinated compounds in the sediment-free transfer cultures that were used for kinetic modeling, 2 mL samples were also taken for DNA extraction. DNA was extracted using a DNeasy PowerSoil Kit (QIAGEN, Hilden, Germany) following the manufacturer’s instructions. The copy number of 16S rRNA genes was determined by real-time quantitative PCR (qPCR) with primers targeting total bacteria (Muyzer et al. 1993) and OHRB including *Dehalococcoides* (Smits et al. 2004), *Geobacter* (Amos et al. 2007), *Dehalogenimonas* (Chen et al. 2014), *Dehalobacter* (Smits et al. 2004), *Desulfitobacterium* (Smits et al. 2004), and *Sulfurospirillum* (Sutton et al. 2015) (Table S1). Assays were performed in triplicates using a CFX384 Real-Time system in a C1000 Thermal Cycler (Bio-Rad Laboratories, USA) with iQ^TM^ SYBR Green Supermix (Bio-Rad Laboratories, USA).

### Analytical methods

Chloroethenes, 1,2-DCA, 1,2-DCP, ethene, and propene were analyzed using a gas chromatograph-mass spectrometer (GC-MS) composed of a Trace GC Ultra (Thermo Fisher Scientific, Waltham, MA, USA) equipped with an Rt®-Q-BOND column (Retek, PA, USA) and a DSQ MS (Thermo Fisher Scientific). Helium was used as a carrier gas with a flow rate of 2 ml min^−1^. The inlet temperature was 100 °C. The split ratio was 30. The temperature program of the column was 40 °C hold for 1 min, followed by an increase at 40 °C min^−1^ to 260 °C and hold for 1.5 min.

### Modeling and parameter estimation

Reductive dechlorination of the chloroethenes in cultures shown in Fig. 1(b) and (d) was modeled using a Michaelis-Menten model with competitive inhibition following Eq. (1)

1 $$ {r}_n=\frac{k_{\max, n}{C}_n}{C_n+{K}_{\mathrm{s},n}\left(1+{\sum}_{i=1}^x\frac{C_{n+\mathrm{i}}}{I_{n+i}}\right)} $$

where *r*_*n*_ (μM d^−1^) is the dechlorination rate that depends on the respective chlorinated compound, *k*_max,*n*_ (μM d^−1^) is the compound-specific maximum utilization rate or degradation constant, *K*_s,*n*_ (μM) is the compound-specific half-velocity constant, *I*_*n*_ (μM) is the compound-specific competitive inhibition rate, and *C*_*n*_ (μM) is the aqueous concentration of the chlorinated compounds. The index *i* represents the number of parent compounds considered in the dechlorination during competitive inhibition that varied with enrichment set-up.

Dihaloelimination of 1,2-DCA to ethene and of 1,2-DCP to propene were also modeled using Eq. (1). In the enrichment cultures containing both chloroethenes/1,2-DCP and 1,2-DCA, competitive inhibition between 1,2-DCA and the other compounds was also included in the models.

As the Michaelis-Menten parameters describing dechlorination are highly correlated, model calibration was performed using AMALGAM (Vrugt and Robinson 2007), a multi-objective, multi-method (ensemble) evolutionary optimization procedure executable in MATLAB. AMALGAM attempts to find for each culture a set of optimal solutions, i.e., an ensemble of optimized kinetic parameters. These solutions have to adhere to the Pareto-principle, which means that all objectives (here, concentrations of the individual dechlorination products) must be met with equal efficiency. The number of Pareto-efficient solutions depends on model complexity and the size of the parameter space as well as the number of model runs and varies for each culture. As such we chose to provide in the results section ranges based on the 50 best parameter combinations of each culture. These were ranked according to their Euclidean distance to the zero-objective point of our *n*-dimensional space, where *n* is the number of culture-specific objectives. Using a compromise solution (Werisch et al. 2014; Wöhling et al. 2008), the parameter set representing the solution with the smallest Euclidean distance on the Pareto front to the zero-objective point was then utilized to create graphical representations as previously outlined (Schneidewind et al. 2014).

All cultures were modeled individually, and models were calibrated on the data obtained from the third spike, during which stable dechlorination patterns were noted. Initially, the cultures with the simplest dechlorination sequences (i.e., VC to ethene or 1,2-DCA to ethene) were modeled, and subsequently model complexity was gradually increased by including additional dechlorination reactions (i.e., cDCE to VC to ethene, etc.). Following this procedure, additional prior information could be used for more complex models to decrease the parameter space (defined by user-set upper and lower boundaries) from which AMALGAM retrieves optimal solutions. Initial boundary values were based on literature (Garant and Lynd 1998; Haest et al. 2010a; Haston and McCarty 1999; Mayer-Blackwell et al. 2016; Schaefer et al. 2009; Schneidewind et al. 2014; Yu and Semprini 2004) for chloroethenes and 1,2-DCA. For 1,2-DCP, boundary conditions were derived from simple Lineweaver-Burk plots (Lineweaver and Burk 1934). Dimensionless, species-dependent Henry coefficients at 20 °C were used to account for volatilization of the chlorinated compounds in the cultures (PCE = 0.711, TCE = 0.419, cDCE = 0.182, VC = 1.075, 1,2-DCA = 0.054, 1,2-DCP = 0.123, ethene = 7.108, propene = 8.923) (Mackay and Shiu 1981; Sander 2015; Staudinger and Roberts 2001).

## Results

### Reductive dechlorination and dynamics of OHRB in the original sediment cultures

**Fig. 2:**
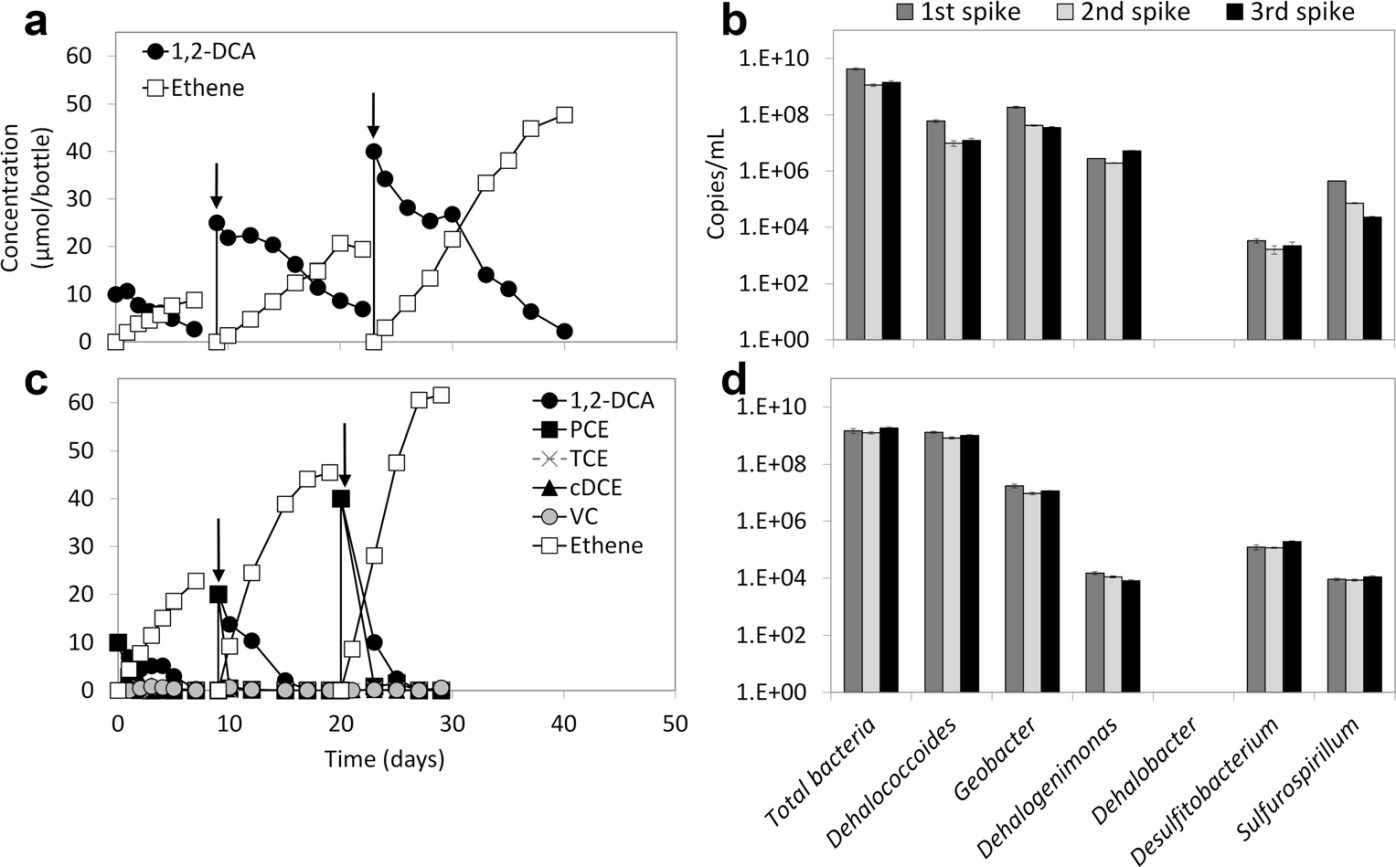
Reductive dechlorination of 1,2-DCA and PCE in the sediment cultures EA (**a**) and EB (**c**) during the last three spikes, and 16S rRNA gene copy numbers of total bacteria, *Dehalococcoides*, *Geobacter*, *Dehalogenimonas*, *Dehalobacter*, *Desulfitobacterium*, and *Sulfurospirillum* at the end of each spike (**b**, **d**). The arrows in panels **a** and **c** indicate re-spike of the chlorinated substrates. Error bars of the qPCR values indicate standard deviations of triplicate qPCRs performed on one sample of each culture

1,2-DCA (10–40 μmol/bottle) was stoichiometrically converted to ethene in the EA and EB sediment cultures without production of any chlorinated intermediates indicating 1,2-DCA dihaloelimination (Fig. 2a). Besides 1,2-DCA, each spike of PCE (10–40 μmol/bottle) in the EB culture was concurrently dechlorinated to ethene (Fig. 2c). *Dehalococcoides* (10^7^–10^8^ 16S rRNA gene copies/mL), *Geobacter* (~ 10^8^ 16S rRNA gene copies/mL), and *Dehalogenimonas* (10^6^–10^7^ 16S rRNA gene copies/mL) were the predominant OHRB in the EA culture (Fig. 2b). In contrast, *Dehalococcoides* (10^8^–10^9^ 16S rRNA gene copies/mL) was the predominant OHRB in the EB culture, and *Geobacter* and *Dehalogenimonas* numbers were ~ 10^7^ and 10^4^ 16S rRNA gene copies/mL, respectively (Fig. 2d). The 16S rRNA gene numbers of *Dehalobacter*, *Desulfitobacterium*, and *Sulfurospirillum* in both EA and EB sediment cultures were below 10^6^ copies/mL, representing less than 0.1% of the total bacterial 16S rRNA gene number (Fig. 2b and d). The 16S rRNA gene copy numbers of OHRB were rather stable during the three spikes of chlorinated compounds (Fig. 2b and d). Hence, for the subsequent sediment-free transfer cultures, qPCR analysis was performed only at the end of the last (third) spike of the chlorinated compound(s).

### Dechlorination and co-contaminant effect of 1,2-DCA and chloroethenes

**Fig. 3:**
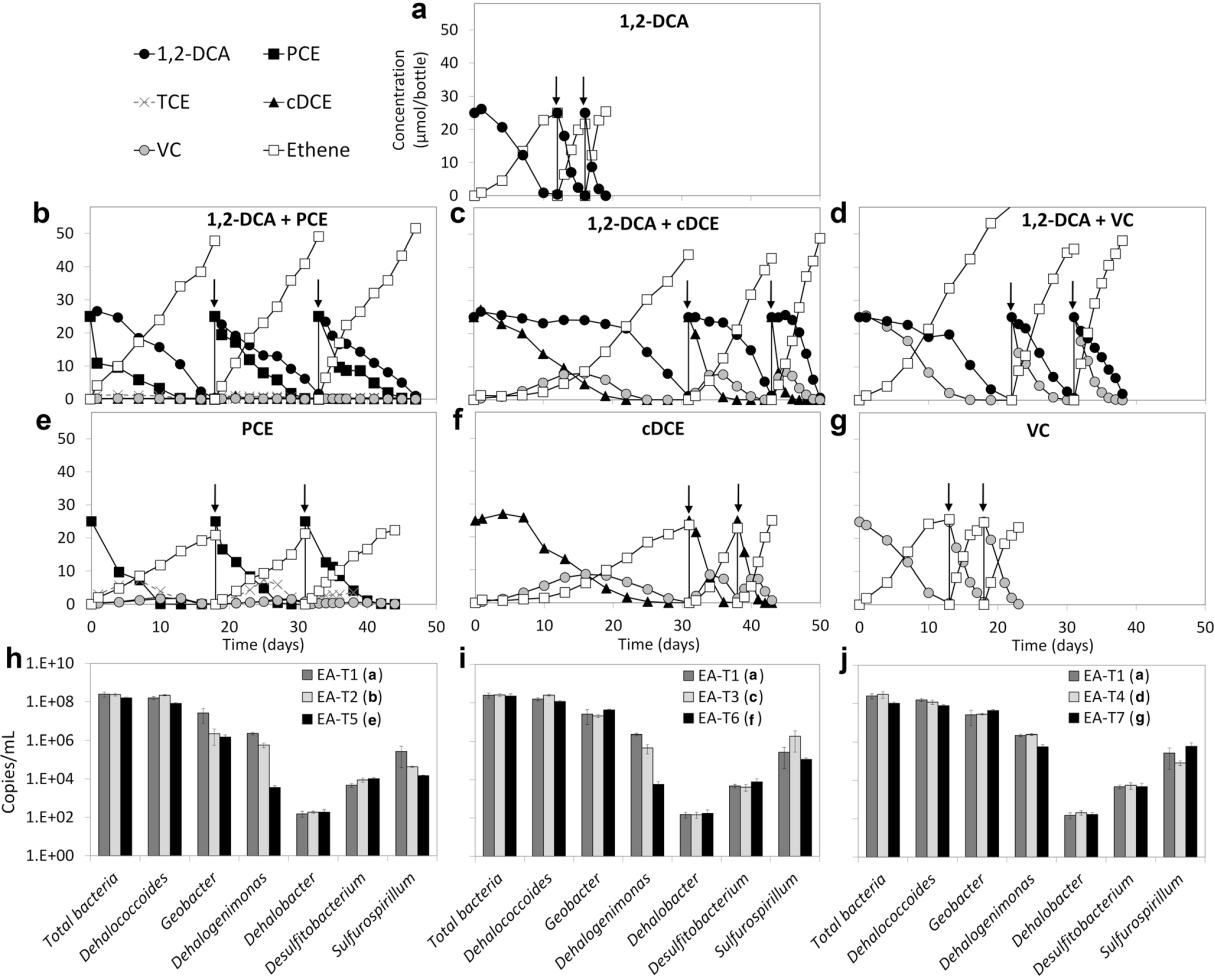
Reductive dechlorination of 1,2-DCA (EA-T1, **a**), 1,2-DCA plus PCE (EA-T2, **b**), 1,2-DCA plus cDCE (EA-T3, **c**), 1,2-DCA plus VC (EA-T4, **d**), PCE (EA-T5, **e**), cDCE (EA-T6, **f**), and VC (EA-T7, **g**) in the sediment-free enrichment cultures obtained from EA sediment culture, and 16S rRNA gene copy numbers of total bacteria, *Dehalococcoides*, *Geobacter*, *Dehalogenimonas*, *Dehalobacter*, *Desulfitobacterium*, and *Sulfurospirillum* at the end of the third spike in these cultures (**h**, **i**, **j**). Each concentration value represents the average measured from duplicate cultures. The arrows in panels **a**–**g** indicate re-spike of the chlorinated substrates. Error bars were not included in panels **a**–**g** for clarity. Error bars of the qPCR values indicate standard deviations of triplicate qPCRs performed on one sample of each of the duplicate cultures (*n* = 2 × 3)

**Fig. 4:**
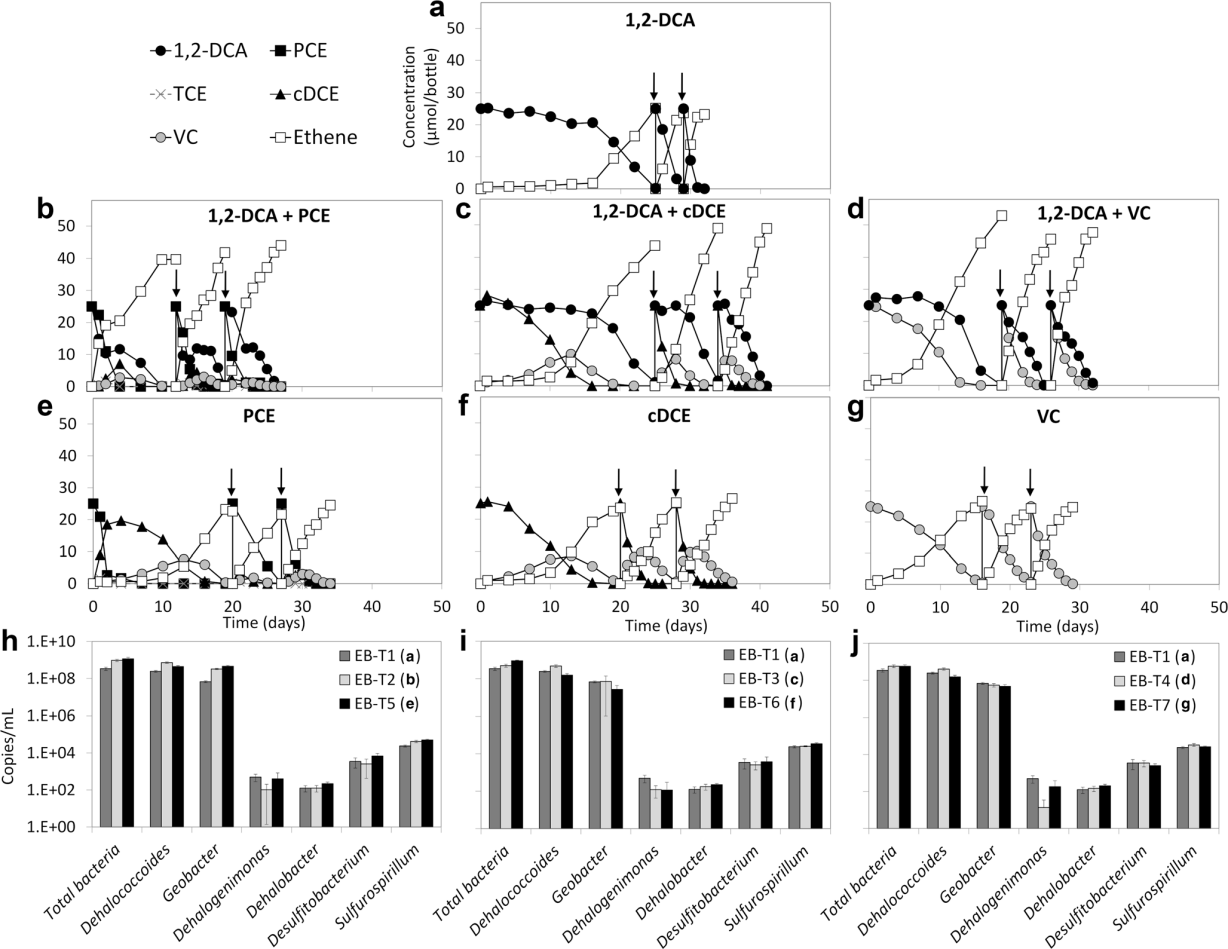
Reductive dechlorination of 1,2-DCA (EB-T1, **a**), 1,2-DCA plus PCE (EB-T2, **b**), 1,2-DCA plus cDCE (EB-T3, **c**), 1,2-DCA plus VC (EB-T4, **d**), PCE (EB-T5, **e**), cDCE (EB-T6, **f**), and VC (EB-T7, **g**) in the sediment-free enrichment cultures obtained from EB sediment culture, and 16S rRNA gene copy numbers of total bacteria, *Dehalococcoides*, *Geobacter*, *Dehalogenimonas*, *Dehalobacter*, *Desulfitobacterium*, and *Sulfurospirillum* at the end of the third spike in these cultures (**h**, **i**, **j**). Each concentration value represents the average measured from duplicate cultures. The arrows in panels **a**–**g** indicate re-spike of the chlorinated substrates. Error bars were not included in panels **a**–**g** for clarity. Error bars of the qPCR values indicate standard deviations of triplicate qPCRs performed on one sample of each of the duplicate cultures (*n* = 2 × 3)

1,2-DCA dechlorination was maintained in the EA and EB sediment-free transfer cultures (cultures EA-T1 and EB-T1, Figs. 3a and 4a). Moreover, PCE, cDCE, and VC were completely dechlorinated to ethene in both EA and EB transfer cultures (cultures EA-T5–T7, Fig. 3e–g, and cultures EB-T5–T7, Fig. 4e–g), although PCE was not amended in the original EA culture. In EA and EB transfer cultures amended with same amounts (25 μmol/bottle each spike) of 1,2-DCA and either PCE, cDCE, or VC, dechlorination of 1,2-DCA was inhibited due to decreased dechlorination rate by the chloroethenes (cultures EA-T2–T4, Fig. 3b–d and cultures EB-T2–T4, Fig. 4b–d), especially when cDCE was present as co-contaminant, where 1,2-DCA dechlorination did not start until cDCE was depleted (culture EA-T3 Fig. 3c and culture EB-T3, Fig. 4c). During dechlorination of the third spike, the time to complete dechlorination of 1,2-DCA increased from around 3 days (Fig. S3–S4) to 7–14 days (Fig. S5–S10) in EA transfer cultures in the presence of chloroethenes, and from around 3 days (Fig. S23–S24) to 6–8 days (Fig. S25–S30) in EB transfer cultures. After the third spike, when the cultures with co-contaminants (Figs. 3b–d and 4b–d) were amended with only 1,2-DCA, its dechlorination was completed in 2–5 days in both EA and EB transfer cultures (Fig. S2A–F), whereas the same amount of 1,2-DCA was dechlorinated in 6–14 days in the presence of chloroethenes. In contrast, no pronounced inhibitory effect of 1,2-DCA on chloroethene dechlorination was observed. Dechlorination of chloroethenes was comparable between cultures where chloroethenes were amended as a single compound (cultures EA-T5–T7, Fig. 3e–g, S11–S16, and cultures EB-T5–T7, Fig. 4e–g, S31–S36) and cultures where they were added together with 1,2-DCA (cultures EA-T2–T4, Fig. 3b–d, S5–S10 and cultures EB-T2–T4, Fig. 4b–d, S25–S30).

The 16S rRNA gene copy number of *Dehalococcoides* in EA transfer cultures (~ 10^8^ copies/mL, Fig. 3h–j) was about one order of magnitude higher than in the original EA sediment culture (Fig. 2b), whereas the 16S rRNA gene copy numbers of *Dehalococcoides* in the EB culture (10^8^–10^9^ copies/mL, Fig. 2d) and its transfer cultures (Fig. 4h–j) were similar. In EA transfer cultures, *Dehalogenimonas* 16S rRNA gene copy numbers were 1–3 orders of magnitude higher in the cultures fed 1,2-DCA, VC, or 1,2-DCA plus chloroethenes (PCE, cDCE, VC) than in the cultures fed only PCE or cDCE (Fig. 3h–j). The 16S rRNA gene copy numbers of *Dehalogenimonas* were below 10^3^ copies/mL in the EB transfer cultures (Fig. 4h–j), in line with the pattern in the EB sediment culture (Fig. 2d). The 16S rRNA gene copy numbers of *Geobacter* in the EA and EB transfer cultures were 10^7^–10^8^ copies/mL (Figs. 3h–j and 4h–j).

### Dechlorination and co-contaminant effect of 1,2-DCA and 1,2-DCP

**Fig. 5:**
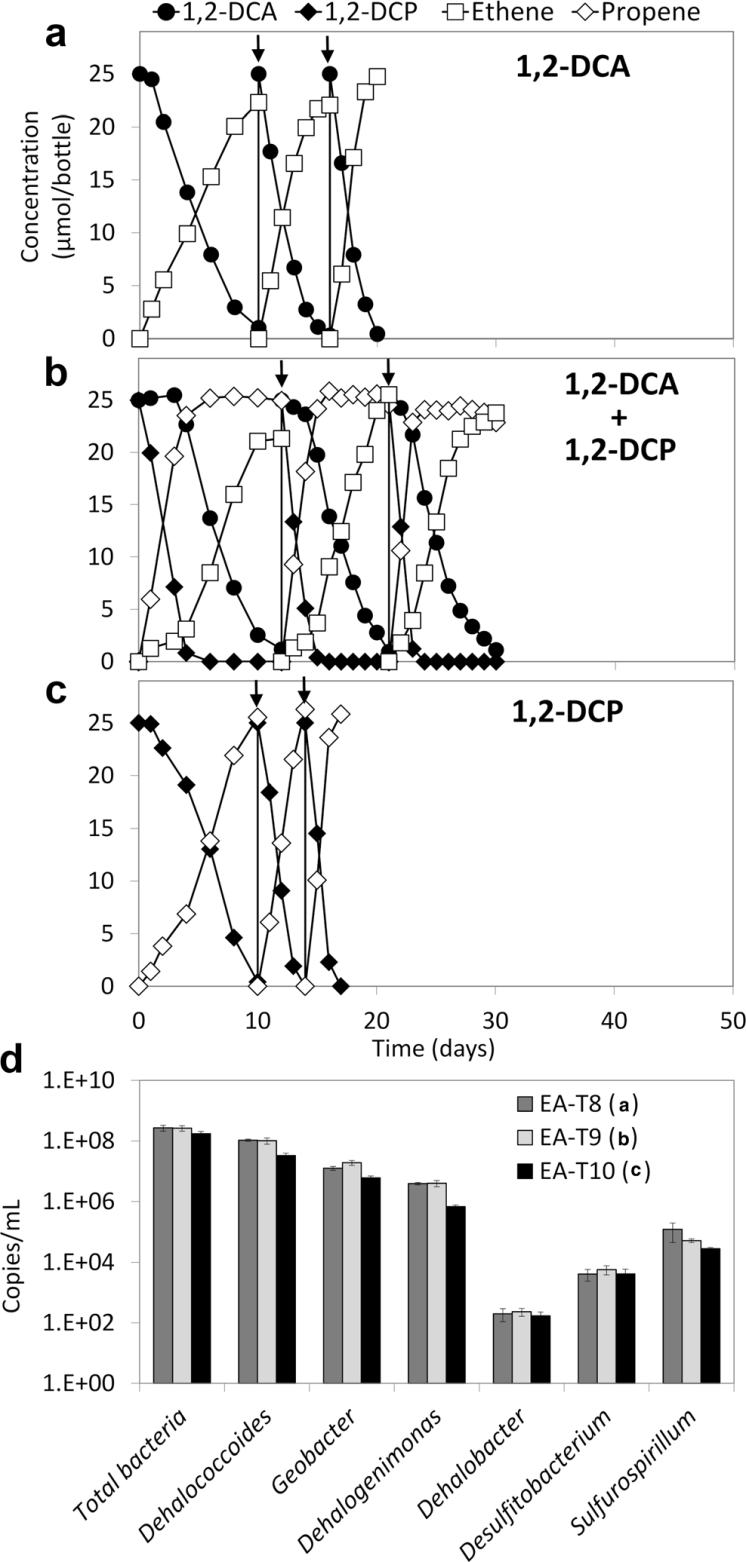
Reductive dechlorination of 1,2-DCA (EA-T8, **a**), 1,2-DCA plus 1,2-DCP (EA-T9, **b**), 1,2-DCP (EA-T10, **c**) in sediment-free cultures derived from the EA transfer culture amended with 1,2-DCP, and 16S rRNA gene copy numbers of total bacteria, *Dehalococcoides*, *Geobacter*, *Dehalogenimonas*, *Dehalobacter*, *Desulfitobacterium*, and *Sulfurospirillum* at the end of the third spike in these cultures (**d**). Each concentration value represents the average measured from duplicate cultures. The arrows in panels **a**–**c** indicate re-spike of the chlorinated substrates. Error bars were not included in panels **a**–**c** for clarity. Error bars of the qPCR values indicate standard deviations of triplicate qPCRs performed on one sample of each of the duplicate cultures (*n* = 2 × 3)

The original EA and EB sediment cultures had not been amended with 1,2-DCP. To study the co-contaminant effect between 1,2-DCA and 1,2-DCP, EA and EB cultures were first transferred (5% inoculum) to fresh media containing only 1,2-DCP (10 μmol/bottle). During 70 days of incubation, more than 90% of the 1,2-DCP in the EA transfer culture was dechlorinated to propene (Fig. S1), whereas no 1,2-DCP dechlorination was observed in the EB transfer culture (data not shown). Therefore, the EA transfer culture fed 1,2-DCP was used to study the co-contaminant effect (Fig. 1d). In the subsequent transfer cultures, 1,2-DCP dechlorination was stably maintained (cultures EA-T10, Fig. 5c), while 1,2-DCA was also dechlorinated (cultures EA-T8, Fig. 5a). Similar to the co-contaminant effect between 1,2-DCA and chloroethenes, 1,2-DCA dechlorination was inhibited in the transfer cultures concurrently amended with 1,2-DCP, whereas no obvious inhibitory effect of 1,2-DCA on 1,2-DCP dechlorination was observed (culture EA-T9, Fig. 5b). Specifically, the time to complete dechlorination of 1,2-DCA was increased from around 3 days (Fig. S17–S18) to 9 days in the presence of 1,2-DCP (Fig. S19–S20), while 1,2-DCP dechlorination was not inhibited (Fig. S19–S22). When these cultures were amended only with 1,2-DCA, its dechlorination was completed in 2 days (Fig. S2G), which was strongly enhanced compared with its dechlorination in the presence of 1,2-DCP (same amount of 1,2-DCA was dechlorinated in 9 days) (Fig. 5b). *Dehalococcoides* (~ 10^8^ 16S rRNA gene copies/mL), *Geobacter* (~ 10^7^ 16S rRNA gene copies/mL), and *Dehalogenimonas* (10^6^–10^7^ 16S rRNA gene copies/mL) were the predominant known OHRB in these transfer cultures, similar to EA transfer cultures amended with 1,2-DCA and chloroethenes (Figs. 3h–g and 5d).

### Dechlorination kinetics

Table 1 provides a summary of parameter ranges for all compounds as compared with the values from previous studies. Those parameter ranges were obtained by modeling dechlorination after the third spike only. Maximum and minimum values of the 50 best parameter combinations for individual cultures are shown in Table S2. *K*_*s*_ and *I* values of the chloroethenes are in the same range as in the previous studies listed in Table 1. A comparison of values of *k*_max_ is less straight forward as many studies provide *k*_max_ in units related to the bacterial cell or protein mass. *k*_max_ estimates for PCE and TCE obtained here are similar to those in Haston and McCarty (1999) and Schneidewind et al. (2014), while *k*_max_ estimates for cDCE and VC in this study were about one order of magnitude higher.

**Table 1:** Range of parameter estimates obtained from modeling using a Michaelis-Menten kinetics approach in comparison with previous studies

Parameter	Unit	This work	Garant and Lynd (1998)^a^	Haston and McCarty (1999)^b^	Yu and Semprini (2004)^c^	Schneidewind et al. (2014)^d^	Mayer-Blackwell et al. (2016)^e^
*k*_max,PCE_	[μM d^−1^]	38.7–443.4	15550^f^	77	12.4/13.3^g^		
*K*_s,PCE_	[μM]	≤ 1.0	70.7	0.1	1.6/3.9		
*I*_PCE_	[μM]	3.7–370	70.7		1.6/3.9		
*k*_max,TCE_	[μM d^−1^]	39.5–1000	9380^f^	59	124/125^g^	2.6–12	
*K*_s,TCE_	[μM]	0.1–14	17.4	1.4	1.8/2.8	2.1–42	
*I*_TCE_	[μM]	3.7–370	17.4		1.8/2.8	3.7–370	
*k*_max,cDCE_	[μM d^−1^]	139.7–245.2	5880^f^	14	13.8/22^g^	0.9–94.4	2688
*K*_s,cDCE_	[μM]	< 0.1–50.8	11.9	3.3	1.8/1.9	3.8–37.8	8.5
*I*_cDCE_	[μM]	3.7–370	11.9		1.8/1.9	3.7–370	9
*k*_max,VC_	[μM d^−1^]	127.4–161.0	6670^f^	13	2.4/8.1^g^	0.4–14.4	
*K*_s,VC_	[μM]	17.7–26.3	383	2.6	62.6/60.2	3.8–37.8	
*I*_VC_	[μM]	3.7–370			62.6/60.2		
*k*_max,DCA_	[μM d^−1^]	< 0.1–7535.8^h^					960
*K*_s,DCA_	[μM]	< 0.1–1032.5^i^					127
*I*_DCA_	[μM]	< 0.1–370^j^					45
*k*_max,DCP_	[μM d^−1^]	220.5–243.1					
*K*_s,DCP_	[μM]	≤ 1.1					
*I*_DCP_	[μM]	1.2−370					

^a^From Table 2 of the reference (competitive case)

^b^From Table 2 of the reference

^c^From Table 2 of the reference, first number EV, second number PM

^d^From Table 2 of the reference

^e^From Table 3 of the reference

^f^μmol/d and g cells

^g^μmol/d and mg protein

^h,i,j^For 1,2-DCA in batches without 1,2-DCP: 157.9 ≤ *k*_max,DCA_ ≤ 1428.9, 2.9 ≤ *K*_s,DCA_ ≤ 1032.5, 2.5 ≤ *I*_DCA_ ≤ 250.0

A comparison of parameter estimates for 1,2-DCP dechlorination was not possible due to the lack of kinetic models in literature. Parameter estimates of 1,2-DCA were comparable with those found by Mayer-Blackwell et al. (2016). However, in our study, they span a rather wide range, defined by cultures EA-T9, where concurrent dechlorination of 1,2-DCA and 1,2-DCP occurred (see Fig. S19 and S20 and Table S2), as further discussed below.

In general, modeled and observed results showed a good fit for cultures amended with a single chlorinated compound (e.g., EA-T1, EA-T7, Figs. S3–S4, S15–S16) indicated by low root-mean-square errors (RMSE, data not shown). Model fits decreased (higher RMSE) for the cultures with more complex reaction networks (e.g., EB-T2, Fig. S25-S26). Duplicate batches showed comparable parameter ranges, and parameters differed by less than one order of magnitude. A notable exception is experiment EA-T9 (Fig. S19, S20) where one of the replicate cultures (culture A) showed relatively narrow parameter ranges for 1,2-DCA, whereas parameter ranges for culture B for 1,2-DCA varied by several orders of magnitude.

## Discussion

The present study revealed inhibition of 1,2-DCA dechlorination in the presence of chloroethenes and 1,2-DCP using organohalide-respiring microbial consortia obtained from a wetland contaminated with agrochemical products. Among the tested chlorinated substrates, cDCE showed the strongest inhibitory effect on 1,2-DCA dechlorination. Dechlorination of 1,2-DCA started only when cDCE was completely depleted (culture EA-T3, Fig. 3c and culture EB-T3, Fig. 4c). This is consistent with previous findings showing that cDCE strongly inhibited 1,2-DCA dechlorination using a continuous enrichment culture containing *Dehalococcoides* (Mayer-Blackwell et al. 2016). We also noted inhibition of 1,2-DCA dechlorination in the presence of PCE, VC, and 1,2-DCP, further supported by the decreased *k*_max,DCA_ and increased *K*_s,DCA_ values compared with the cultures amended with 1,2-DCA only. Notably, the inhibitory pattern of PCE, VC, and 1,2-DCP on 1,2-DCA dechlorination was different from that of cDCE. In cultures containing 1,2-DCA with either PCE, VC, or 1,2-DCP as co-contaminants, the cultures concurrently dechlorinated both amended chlorinated compounds. However, delayed dechlorination of 1,2-DCA due to a decreased dechlorination rate was observed during concurrent dechlorination of 1,2-DCA and PCE (culture EA-T2, Fig. 3b and culture EB-T2, Fig. 4b), probably because of the cDCE production from PCE.

The observed inhibitory effect of VC on 1,2-DCA dechlorination is in contrast to what was reported by Mayer-Blackwell et al. (2016) using a continuous enrichment culture containing *Dehalococcoides* where VC had a negligible inhibitory effect on 1,2-DCA dechlorination. Interestingly, Mayer-Blackwell et al. (2016) found that long-term exposure of the continuous culture to 1,2-DCA shifted the *Dehalococcoides* population, leading to diminished VC dechlorinating ability. In contrast, we found no inhibitory effect of 1,2-DCA on the dechlorination of chloroethenes and 1,2-DCP in batch cultures.

Based on the data presented here, it is likely that the original sediments in EA and EB cultures contained at least two different *Dehalococcoides* populations. The 1,2-DCP dechlorinating population was maintained during enrichment in the presence of 1,2-DCA (EA culture) but likely lost during incubation in the presence of 1,2-DCA plus PCE (EB culture). This suggests that 1,2-DCA but not PCE was also a growth substrate for the 1,2-DCP dechlorinating *Dehalococcoides* population, whereas PCE was likely a substrate for the other *Dehalococcoides* population. The EA transfer cultures were also able to dechlorinate PCE (Fig. 3b, e). This indicates that feeding 1,2-DCA alone in the EA sediment culture also maintained the *Dehalococcoides* population capable of PCE dechlorination, and therefore, 1,2-DCA was also a growth substrate for the PCE-dechlorinating *Dehalococcoides* population. Feeding 1,2-DCA plus PCE likely promoted selective growth of the 1,2-DCA/PCE-dechlorinating *Dehalococcoides* over the 1,2-DCA/1,2-DCP dechlorinating *Dehalococcoides*. This is also likely the reason for the loss of *Dehalogenimonas* in the EB culture, as *Dehalogenimonas* is known to dechlorinate 1,2-DCA and 1,2-DCP via dihaloelimination but does not dechlorinate PCE (Bowman et al. 2013; Moe et al. 2009). Accordingly, *Dehalogenimonas* was only maintained in the EA transfer cultures amended with substrates known to support *Dehalogenimonas* growth (e.g., 1,2-DCA or 1,2-DCA plus VC or 1,2-DCA plus 1,2-DCP) (Maness et al. 2012; Martín-González et al. 2015; Moe et al. 2016; Yang et al. 2017) (Fig. 3h–j). Notably, feeding solely PCE or cDCE that are not known growth substrates for *Dehalogenimonas* decreased *Dehalogenimonas* 16S rRNA gene copy numbers by 1–3 orders of magnitude (Fig. 3h, i), whereas feeding solely VC or 1,2-DCP that are known substrates to support *Dehalogenimonas* growth (Bowman et al. 2013; Moe et al. 2009; Yang et al. 2017) did not strongly reduce its 16S rRNA gene copy numbers (Figs. 3j and 4d). *Geobacter*, which is known to dechlorinate PCE/TCE (Sung et al. 2006), and 1,2-DCA (Duhamel and Edwards 2007), was stably maintained in both EA (Fig. 3h–j) and EB (Fig. 4h–j) sediment-free transfer cultures. Our results indicate that the type of chlorinated substrate drives the selection of OHRB. Likewise, a recent study showed that a *Dehalococcoides* population shift was driven by different chlorinated electron acceptors in enrichment cultures containing *Dehalococcoides* and at a contaminated site (Pérez-de-Mora et al. 2018).

Michaelis-Menten kinetics was successfully used to model dechlorination after the third spike when dechlorination followed a concave pattern. Parameter estimates obtained here compare well with those obtained in previous studies (see Table 1). Different kinetic models could be more suited to model dechlorination after the first and the second spikes. Especially dechlorination after the first spike might be modeled more successfully using Monod kinetics (see also Schneidewind et al., 2014) to better account for an apparent lag phase before the onset of dechlorination. However, Monod modeling was not used in this study due to limited information on microbial interactions (growth and decay patterns).

An interpretation of competitive inhibition is not straightforward from the obtained inhibition constants *I* (Table S2). For example, cDCE seems to have a pronounced effect on 1,2-DCA dechlorination only at starting concentrations of cDCE (~*I*_*cDCE*_). As soon as cDCE concentrations drop by a factor ≥ 10, inhibition of 1,2-DCA dechlorination by cDCE becomes much less important. The calibrated inhibition constants suggest that VC is a stronger inhibitor on 1,2-DCA dechlorination than cDCE in all cultures except EB-T2 (*I*_*VC*_ is substantially below *I*_*cDCE*_). However, *I*_*VC*_ is largest in cultures EA-T4 and EB-T4 where no higher chlorinated compounds were present. A higher competitive inhibition constant indicates a smaller inhibitive effect. The parameter for VC probably lumps inhibition effects from higher chlorinated parent products when present, resulting in a higher simulated inhibition effect of VC in mixed compound tests.

In addition, interpreting parameter estimates (especially inhibition constants) obtained from cultures with multiple chlorinated compounds proved challenging, as strong cross-correlation exists among the parameters of the Michaelis-Menten kinetics. For example, we observed comparable *K*_s_ and *k*_max_ values for chloroethenes and 1,2-DCP in cultures with and without 1,2-DCA. In contrast, *k*_max,DCA_ values decreased and *K*_s,DCA_ values increased in multi-compound cultures compared with the cultures amended with 1,2-DCA only. This hints towards an effect of the chloroethenes and 1,2-DCP on 1,2-DCA dechlorination. However, the best parameter combination did not put this effect in the inhibition constants, but rather in the dechlorination constants of 1,2-DCA itself. Another example is culture EA-T9: a small *K*_s_ of 1,2-DCA is counteracted by a small inhibition constant of 1,2-DCP in the duplicate cultures. In other words, the obtained parameters suggest that dechlorination of 1,2-DCA could occur at a maximal rate at low substrate concentration, but would then be more inhibited by 1,2-DCP; or in contrast, the maximal dechlorination rate of 1,2-DCA is not attained under the current experimental conditions but its dechlorination would be less inhibited by 1,2-DCP. However, both parameter combinations would yield a proper fit of the experimental observations, proving the non-uniqueness of the solution (Beven 2001).

The use of results from less complex culture set-ups in the refinement of the parameter space, from which AMALGAM choses viable solutions for more complex set-ups (e.g., EA-T1, EA-T4 and EA-T6 for EA-T2), allowed us to reduce the uncertainty on the parameter estimates. However, the problem of non-uniqueness still remains, e.g., the existence of multiple parameter combinations producing an equally good fit. Uncertainty on the parameter estimates arises due to incomplete or insufficient information on the dechlorination processes in the individual cultures (e.g., information on microbial interactions or on necessary micro-nutrients).

In conclusion, the inhibitory effect of chloroethenes and 1,2-DCP on 1,2-DCA dechlorination identified in this study has important implications for understanding the persistence of 1,2-DCA at many contaminated sites. For effective bioremediation of such contaminated sites, it will be necessary to first remove potential inhibitors such as cDCE as well as its parent compounds PCE and TCE, which can delay 1,2-DCA dechlorination and even cause loss of 1,2-DCA/1,2-DCP dechlorinating *Dehalococcoides* and *Dehalogenimonas* populations. Further studies are needed to better understand the inhibitory mechanisms. Possible experimental approaches include identification of the genes and enzymes involved in 1,2-DCA dichlorination, study the transcriptional regulation of these genes, and assess potential competitive inhibition of the enzymes. Theoretical modeling experiments that more rigorously look into the effect of the size of the parameter space or the use of different estimation algorithms could further improve our understanding of parameter/model uncertainty.

## Electronic supplementary material

ESM 1 (PDF 2535 kb)

ESM 2 (XLSX 24 kb)

## References

[CR1] Amos BK, Sung Y, Fletcher KE, Gentry TJ, Wu W-M, Criddle CS, Zhou J, Löffler FE (2007). Detection and quantification of *Geobacter lovleyi* strain SZ: implications for bioremediation at tetrachloroethene-and uranium-impacted sites. Appl Environ Microbiol.

[CR2] Atashgahi S, Lu Y, Smidt H, Adrian L, Löffler FE (2016). Overview of known organohalide-respiring bacteria—phylogenetic diversity and environmental distribution. Organohalide-respiring bacteria.

[CR3] Atashgahi S, Lu Y, Zheng Y, Saccenti E, Suarez-Diez M, Ramiro-Garcia J, Eisenmann H, Elsner M, Stams AJM, Springael D, Dejonghe W, Smidt H (2017). Geochemical and microbial community determinants of reductive dechlorination at a site biostimulated with glycerol. Environ Microbiol.

[CR4] Atashgahi S, Sanchez-Andrea I, Heipieper HJ, van der Meer JR, Stams AJM, Smidt H (2018). Prospects for harnessing biocide resistance for bioremediation and detoxification. Science.

[CR5] Beven K (2001). How far can we go in distributed hydrological modelling?. Hydrol Earth Syst Sci.

[CR6] Bowman KS, Nobre MF, da Costa MS, Rainey FA, Moe WM (2013). *Dehalogenimonas alkenigignens* sp. nov., a chlorinated-alkane-dehalogenating bacterium isolated from groundwater. Int J Syst Evol Microbiol.

[CR7] Brovelli A, Barry DA, Robinson C, Gerhard JI (2012). Analysis of acidity production during enhanced reductive dechlorination using a simplified reactive transport model. Adv Water Resour.

[CR8] Carvalho M, Ferreira Jorge R, Pacheco C, De Marco P, Castro P (2005). Isolation and properties of a pure bacterial strain capable of fluorobenzene degradation as sole carbon and energy source. Environ Microbiol.

[CR9] Chambon JC, Bjerg PL, Scheutz C, Bælum J, Jakobsen R, Binning PJ (2013). Review of reactive kinetic models describing reductive dechlorination of chlorinated ethenes in soil and groundwater. Biotechnol Bioeng.

[CR10] Chan WW, Grostern A, Löffler FE, Edwards EA (2011). Quantifying the effects of 1,1,1-trichloroethane and 1,1-dichloroethane on chlorinated ethene reductive dehalogenases. Environ Sci Technol.

[CR11] Chen J, Bowman KS, Rainey FA, Moe WM (2014). Reassessment of PCR primers targeting 16S rRNA genes of the organohalide-respiring genus *Dehalogenimonas*. Biodegradation.

[CR12] Christ JA, Abriola LM (2007). Modeling metabolic reductive dechlorination in dense non-aqueous phase liquid source-zones. Adv Water Resour.

[CR13] Colombani N, Pantano A, Mastrocicco M, Petitta M (2014). Reactive modelling of 1,2-DCA and DOC near the shoreline. J Contam Hydrol.

[CR14] Corapcioglu MY, Sung K, Kim J (2004). Parameter determination of sequential reductive dehalogenation reactions of chlorinated hydrocarbons. Transport Porous Med.

[CR15] Cupples AM, Spormann AM, McCarty PL (2004). Vinyl chloride and *cis*-dichloroethene dechlorination kinetics and microorganism growth under substrate limiting conditions. Environ Sci Technol.

[CR16] Da Silva ML, Alvarez P (2008). Exploring the correlation between halorespirer biomarker concentrations and TCE dechlorination rates. J Environ Eng.

[CR17] Dillehay JL, Bowman KS, Yan J, Rainey FA, Moe WM (2014). Substrate interactions in dehalogenation of 1,2-dichloroethane, 1,2-dichloropropane, and 1,1,2-trichloroethane mixtures by *Dehalogenimonas* spp. Biodegradation.

[CR18] Duhamel M, Edwards EA (2006). Microbial composition of chlorinated ethene-degrading cultures dominated by *Dehalococcoides*. FEMS Microbiol Ecol.

[CR19] Duhamel M, Edwards EA (2007). Growth and yields of dechlorinators, acetogens, and methanogens during reductive dechlorination of chlorinated ethenes and dihaloelimination of 1,2-dichloroethane. Environ Sci Technol.

[CR20] Edwards EA (2014). Breathing the unbreathable. Science.

[CR21] Ellis DE, Lutz EJ, Odom JM, Buchanan RJ, Bartlett CL, Lee MD, Harkness MR, DeWeerd KA (2000). Bioaugmentation for accelerated in situ anaerobic bioremediation. Environ Sci Technol.

[CR22] EPA (2018). 2018 Edition of the drinking water standards and health advisories tables.

[CR23] Field JA, Sierra-Alvarez R (2004). Biodegradability of chlorinated solvents and related chlorinated aliphatic compounds. Rev Environ Sci Biotechnol.

[CR24] Garant H, Lynd L (1998). Applicability of competitive and noncompetitive kinetics to the reductive dechlorination of chlorinated ethenes. Biotechnol Bioeng.

[CR25] Grostern A, Edwards EA (2009). Characterization of a *Dehalobacter* coculture that dechlorinates 1,2-dichloroethane to ethene and identification of the putative reductive dehalogenase gene. Appl Environ Microbiol.

[CR26] Grostern A, Chan WW, Edwards EA (2009). 1,1,1-Trichloroethane and 1,1-dichloroethane reductive dechlorination kinetics and co-contaminant effects in a *Dehalobacter*-containing mixed culture. Environ Sci Technol.

[CR27] Haest PJ, Springael D, Smolders E (2010). Dechlorination kinetics of TCE at toxic TCE concentrations: assessment of different models. Water Res.

[CR28] Haest PJ, Springael D, Smolders E (2010). Modelling reactive CAH transport using batch experiment degradation kinetics. Water Res.

[CR29] Haston ZC, McCarty PL (1999). Chlorinated ethene half-velocity coefficients (Ks) for reductive dehalogenation. Environ Sci Technol.

[CR30] Kouznetsova I, Mao X, Robinson C, Barry DA, Gerhard JI, McCarty PL (2010). Biological reduction of chlorinated solvents: batch-scale geochemical modeling. Adv Water Resour.

[CR31] Lai Y, Becker JG (2013). Compounded effects of chlorinated ethene inhibition on ecological interactions and population abundance in a *Dehalococcoides-Dehalobacter* coculture. Environ Sci Technol.

[CR32] Lineweaver H, Burk D (1934). The determination of enzyme dissociation constants. J Am Chem Soc.

[CR33] Low A, Zhao S, Rogers MJ, Zemb O, Lee M, He J, Manefield M (2019) Isolation, characterization and bioaugmentation of an acidotolerant 1,2-dichloroethane respiring *Desulfitobacterium* species from a low pH aquifer. FEMS Microbiol Ecol 95(5):fiz055. 10.1093/femsec/fiz055 10.1093/femsec/fiz055 30980656

[CR34] Mackay D, Shiu WY (1981). A critical review of Henry’s law constants for chemicals of environmental interest. J Phys Chem Ref Data.

[CR35] Malaguerra F, Chambon JC, Bjerg PL, Scheutz C, Binning PJ (2011). Development and sensitivity analysis of a fully kinetic model of sequential reductive dechlorination in groundwater. Environ Sci Technol.

[CR36] Maness AD, Bowman KS, Yan J, Rainey FA, Moe WM (2012). *Dehalogenimonas* spp. can reductively dehalogenate high concentrations of 1,2-dichloroethane, 1,2-dichloropropane, and 1,1,2-trichloroethane. AMB Express.

[CR37] Martín-González L, Hatijah Mortan S, Rosell M, Parladé E, Martínez-Alonso M, Gaju N, Caminal G, Adrian L, Marco-Urrea E (2015). Stable carbon isotope fractionation during 1,2-dichloropropane-to-propene transformation by an enrichment culture containing *Dehalogenimonas* strains and a *dcpA* gene. Environ Sci Technol.

[CR38] Marzorati M, De Ferra F, Van Raemdonck H, Borin S, Allifranchini E, Carpani G, Serbolisca L, Verstraete W, Boon N, Daffonchio D (2007). A novel reductive dehalogenase, identified in a contaminated groundwater enrichment culture and in *Desulfitobacterium dichloroeliminans* strain DCA1, is linked to dehalogenation of 1,2-dichloroethane. Appl Environ Microbiol.

[CR39] Mayer-Blackwell K, Fincker M, Molenda O, Callahan B, Sewell H, Holmes S, Edwards EA, Spormann AM (2016). 1,2-Dichloroethane exposure alters the population structure, metabolism, and kinetics of a trichloroethene-dechlorinating dehalococcoides mccartyi consortium. Environ Sci Technol.

[CR40] Maymó-Gatell X, Anguish T, Zinder SH (1999). Reductive dechlorination of chlorinated ethenes and 1,2-dichloroethane by “*Dehalococcoides ethenogenes*” 195. Appl Environ Microbiol.

[CR41] Meckenstock RU, Elsner M, Griebler C, Lueders T, Stumpp C, Aamand J, Agathos SN, Albrechtsen H-J, Bastiaens L, Bjerg PL (2015). Biodegradation: updating the concepts of control for microbial cleanup in contaminated aquifers. Environ Sci Technol.

[CR42] Moe WM, Yan J, Nobre MF, da Costa MS, Rainey FA (2009). *Dehalogenimonas lykanthroporepellens* gen. nov., sp. nov., a reductively dehalogenating bacterium isolated from chlorinated solvent-contaminated groundwater. Int J Syst Evol Microbiol.

[CR43] Moe WM, Rainey FA, Yan J, Adrian L, Löffler FE (2016). The genus *Dehalogenimonas*. Organohalide-respiring bacteria.

[CR44] Muyzer G, De Waal EC, Uitterlinden AG (1993). Profiling of complex microbial populations by denaturing gradient gel electrophoresis analysis of polymerase chain reaction-amplified genes coding for 16S rRNA. Appl Environ Microbiol.

[CR45] Parthasarathy A, Stich TA, Lohner ST, Lesnefsky A, Britt RD, Spormann AM (2015) Biochemical and EPR-spectroscopic investigation into heterologously expressed vinyl chloride reductive dehalogenase (VcrA) from *Dehalococcoides mccartyi* strain VS. J Am Chem Soc 137(10):3525–3532. 10.1021/ja511653d 10.1021/ja511653d PMC4516053 25686300

[CR46] Pérez-de-Mora A, Lacourt A, McMaster ML, Liang X, Dworatzek SM, Edwards EA (2018). Chlorinated electron acceptor abundance drives selection of *Dehalococcoides mccartyi* (*D. mccartyi*) strains in dechlorinating enrichment cultures and groundwater environments. Front Microbiol.

[CR47] Sander R (2015). Compilation of Henry’s law constants (version 4.0) for water as solvent. Atmos Chem Phys.

[CR48] Schaefer CE, Condee CW, Vainberg S, Steffan RJ (2009). Bioaugmentation for chlorinated ethenes using *Dehalococcoides* sp.: comparison between batch and column experiments. Chemosphere.

[CR49] Schneidewind U, Haest PJ, Atashgahi S, Maphosa F, Hamonts K, Maesen M, Calderer M, Seuntjens P, Smidt H, Springael D, Dejonghe W (2014) Kinetics of dechlorination by *Dehalococcoides mccartyi* using different carbon sources. J Contam Hydrol 157:25–36. 10.1016/j.jconhyd.2013.10.006 10.1016/j.jconhyd.2013.10.006 24275111

[CR50] Smits TH, Devenoges C, Szynalski K, Maillard J, Holliger C (2004). Development of a real-time PCR method for quantification of the three genera *Dehalobacter, Dehalococcoides*, and *Desulfitobacterium* in microbial communities. J Microbiol Methods.

[CR51] Stams AJ, Van Dijk JB, Dijkema C, Plugge CM (1993). Growth of syntrophic propionate-oxidizing bacteria with fumarate in the absence of methanogenic bacteria. Appl Environ Microbiol.

[CR52] Staudinger J, Roberts PV (2001). A critical compilation of Henry’s law constant temperature dependence relations for organic compounds in dilute aqueous solutions. Chemosphere.

[CR53] Sung Y, Fletcher KE, Ritalahti KM, Apkarian RP, Ramos-Hernández N, Sanford RA, Mesbah NM, Löffler FE (2006). *Geobacter lovleyi* sp. nov. strain SZ, a novel metal-reducing and tetrachloroethene-dechlorinating bacterium. Appl Environ Microbiol.

[CR54] Sutton NB, Atashgahi S, Saccenti E, Grotenhuis T, Smidt H, Rijnaarts HH (2015). Microbial community response of an organohalide respiring enrichment culture to permanganate oxidation. PLoS One.

[CR55] Vandermaesen J, Horemans B, Bers K, Vandermeeren P, Herrmann S, Sekhar A, Seuntjens P, Springael D (2016). Application of biodegradation in mitigating and remediating pesticide contamination of freshwater resources: state of the art and challenges for optimization. Appl Microbiol Biotechnol.

[CR56] Vrugt JA, Robinson BA (2007). Improved evolutionary optimization from genetically adaptive multimethod search. Proc Natl Acad Sci.

[CR57] Wang S, He J (2013). Dechlorination of commercial PCBs and other multiple halogenated compounds by a sediment-free culture containing *Dehalococcoides* and *Dehalobacter*. Environ Sci Technol.

[CR58] Weatherill JJ, Atashgahi S, Schneidewind U, Krause S, Ullah S, Cassidy N, Rivett MO (2018). Natural attenuation of chlorinated ethenes in hyporheic zones: a review of key biogeochemical processes and in-situ transformation potential. Water Res.

[CR59] Werisch S, Grundmann J, Al-Dhuhli H, Algharibi E, Lennartz F (2014). Multiobjective parameter estimation of hydraulic properties for a sandy soil in Oman. Environ Earth Sci.

[CR60] Wöhling T, Vrugt JA, Barkle GF (2008). Comparison of three multiobjective optimization algorithms for inverse modeling of vadose zone hydraulic properties. Soil Sci Soc Am J.

[CR61] Yang Y, Higgins SA, Yan J, Şimşir B, Chourey K, Iyer R, Hettich RL, Baldwin B, Ogles DM, Löffler FE (2017). Grape pomace compost harbors organohalide-respiring *Dehalogenimonas* species with novel reductive dehalogenase genes. ISME J.

[CR62] Yu S, Semprini L (2004). Kinetics and modeling of reductive dechlorination at high PCE and TCE concentrations. Biotechnol Bioeng.

[CR63] Yu S, Dolan ME, Semprini L (2005). Kinetics and inhibition of reductive dechlorination of chlorinated ethylenes by two different mixed cultures. Environ Sci Technol.

[CR64] Yu R, Peethambaram HS, Falta RW, Verce MF, Henderson JK, Bagwell CE, Brigmon RL, Freedman DL (2013). Kinetics of 1,2-dichloroethane and 1,2-dibromoethane biodegradation in anaerobic enrichment cultures. Appl Environ Microbiol.

