# Supplementary material for: Reductive dechlorination of 1,2-dichloroethane in the presence of chloroethenes and 1,2-dichloropropane as co-contaminants
Source: Appl Microbiol Biotechnol. 2019 Jun 28;103(16):6837–49. doi: 10.1007/s00253-019-09985-8 (PMC6667407; doi:10.1007/s00253-019-09985-8)
Supplement: Supplementary file 1 — (PDF 2535 kb) [file 253_2019_9985_MOESM1_ESM.pdf]

Applied Microbiology and Biotechnology

Reductive dechlorination of 1,2-dichloroethane in the presence of chloroethenes and 1,2-dichloropropane as co-contaminants

Peng Peng[1#], Uwe Schneidewind[2#], Pieter Jan Haest[3,4], Tom N.P. Bosma[1], Anthony S. Danko[5], Hauke Smidt[1] and Siavash Atashgahi[1*]

[1] Laboratory of Microbiology, Wageningen University & Research, Wageningen, The Netherlands

[2] Department of Civil and Environmental Engineering, Western University, London, ON, Canada

[3] Advanced Groundwater Techniques (AGT), Aartselaar, Belgium

[4] Division of Geology, Department of Earth and Environmental Sciences, KU Leuven, Heverlee, Belgium

[5] Centre for Natural Resources and the Environment (CERENA), Department of Mining Engineering, University of Porto (FEUP), Porto, Portugal

[#]equal contribution

[*]Correspondence: Siavash Atashgahi, siavash.atashgahi@wur.nl

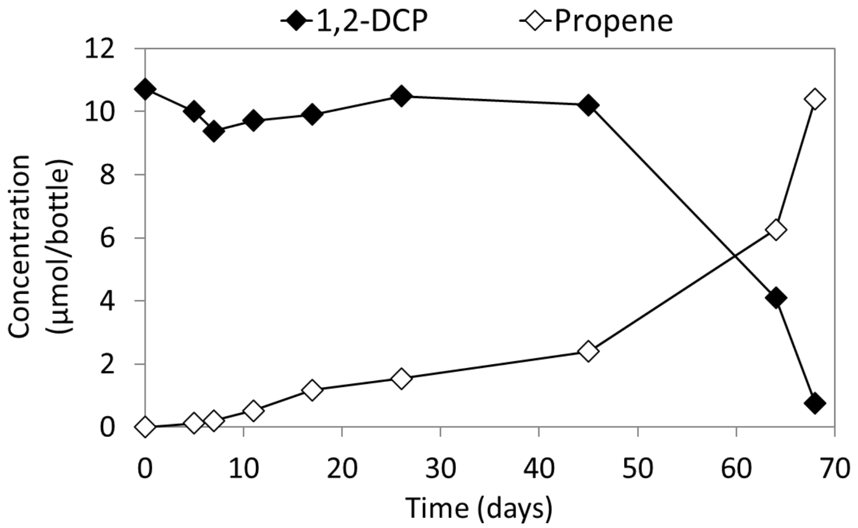

Fig. S1: Reductive dechlorination of 1,2-DCP in EA transfer culture.

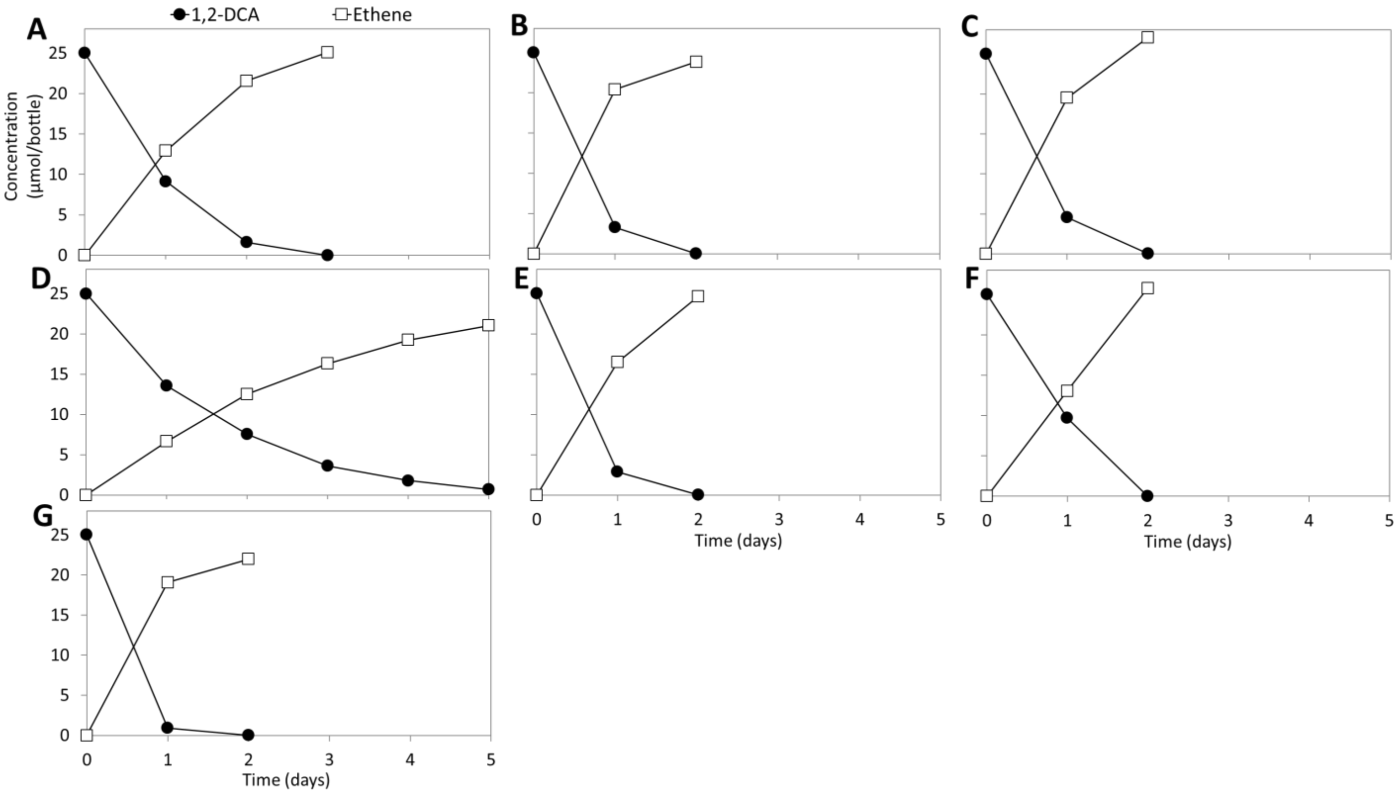

Fig. S2: Reductive dechlorination of 1,2-DCA alone in EA and EB transfer cultures previously amended with 1,2-DCA plus PCE (cultures EA-T2 and EB-T2) (A and D); 1,2-DCA plus cDCE (cultures EA-T3 and EB-T3) (B and E); 1,2-DCA plus VC (cultures EA-T4 and EB-T4) (C and F); 1,2-DCA plus 1,2-DCP (culture EA-T9) (G). Each concentration value represents the average measurement of duplicate cultures.

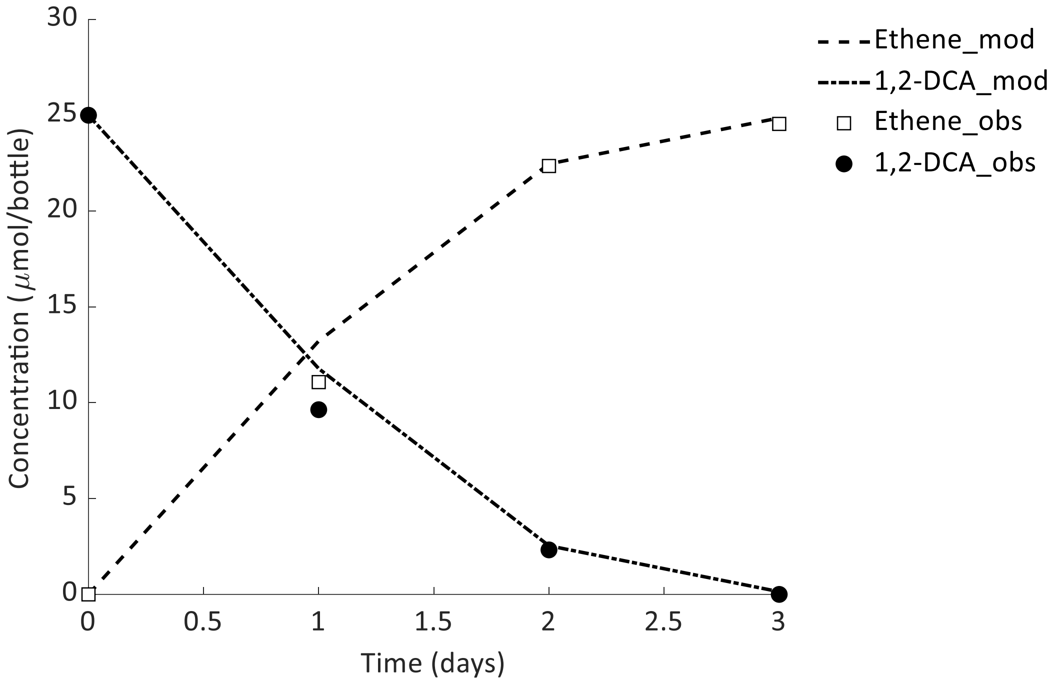

Fig. S3: Modeled (mod) and observed (obs) concentrations in culture EA-T1_A.

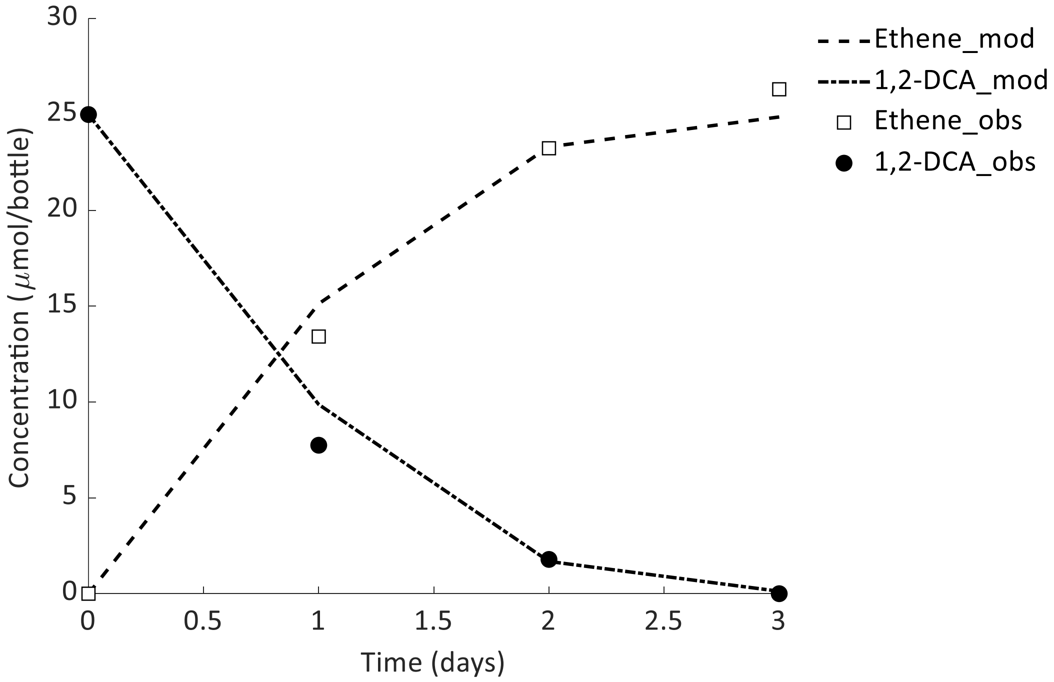

Fig. S4: Modeled (mod) and observed (obs) concentrations in culture EA-T1_B.

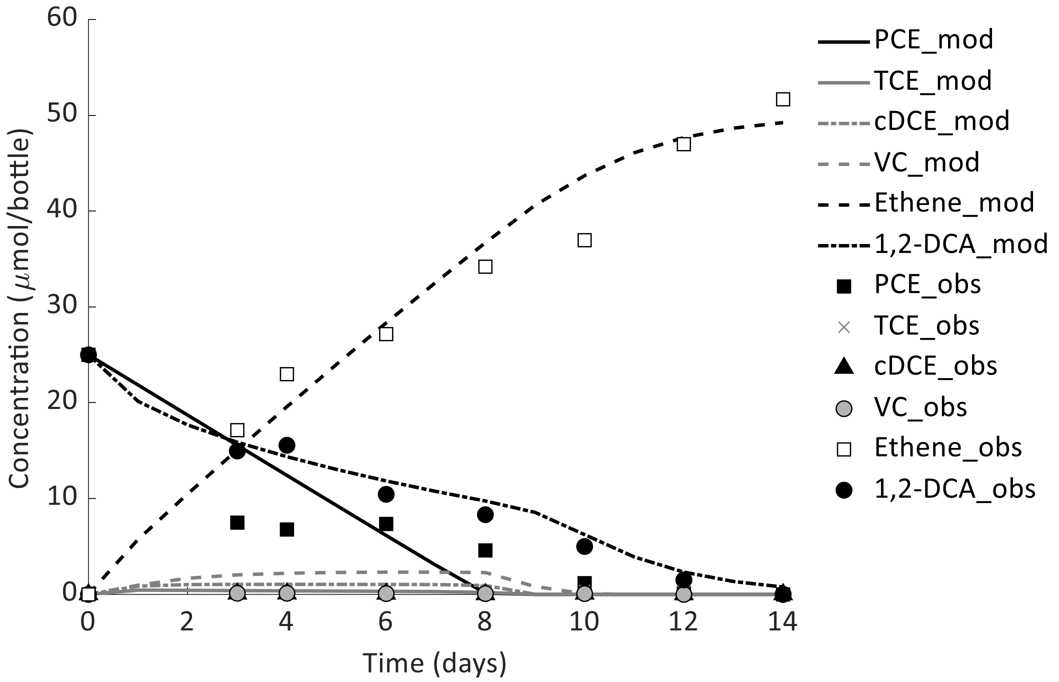

Fig. S5: Modeled (mod) and observed (obs) concentrations in culture EA-T2_A.

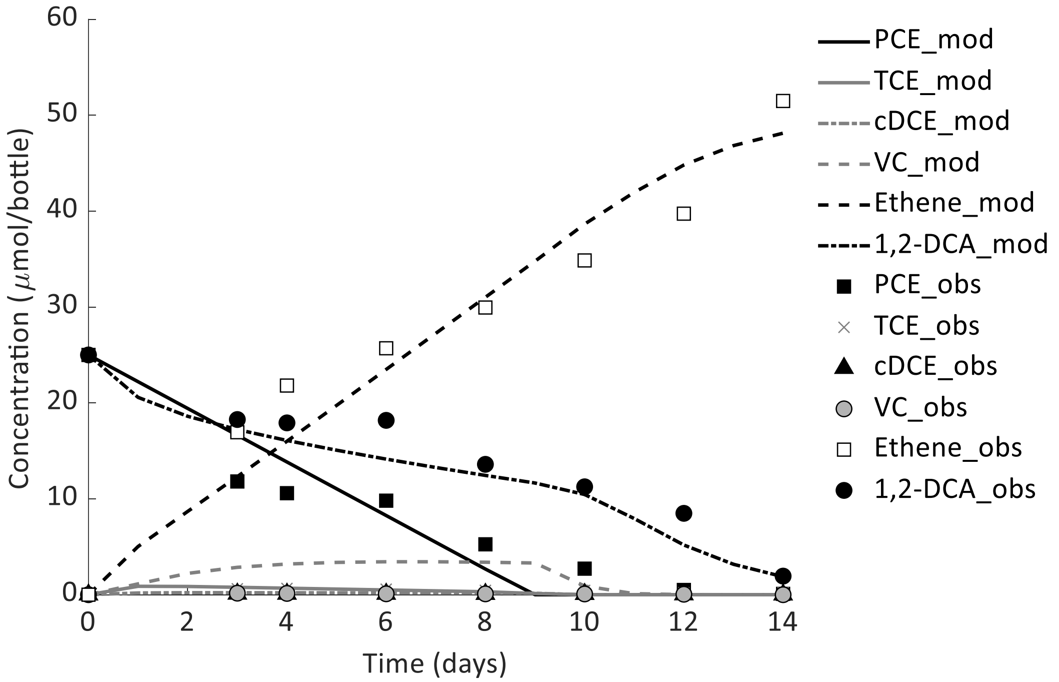

Fig. S6: Modeled (mod) and observed (obs) concentrations in culture EA-T2_B.

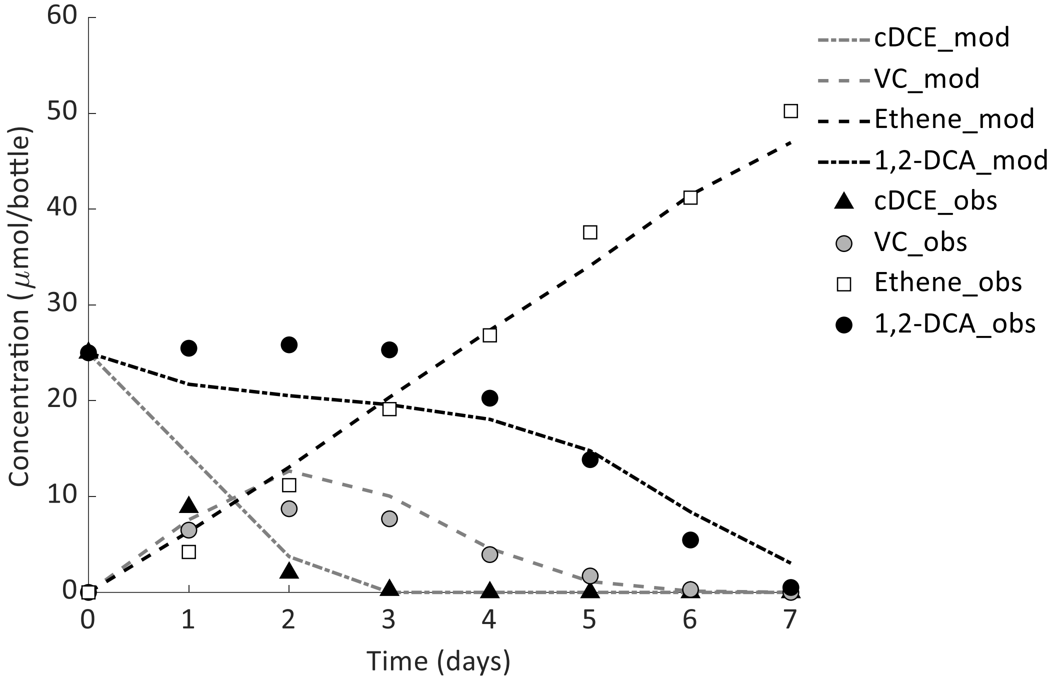

Fig. S7: Modeled (mod) and observed (obs) concentrations in culture EA-T3_A.

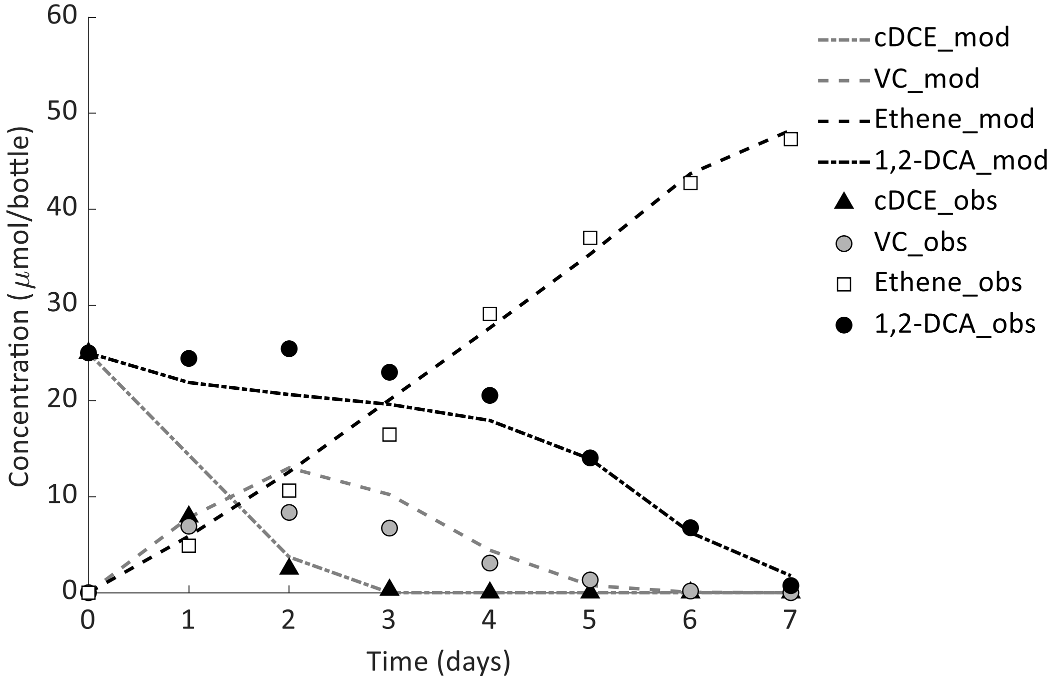

Fig. S8: Modeled (mod) and observed (obs) concentrations in culture EA-T3_B.

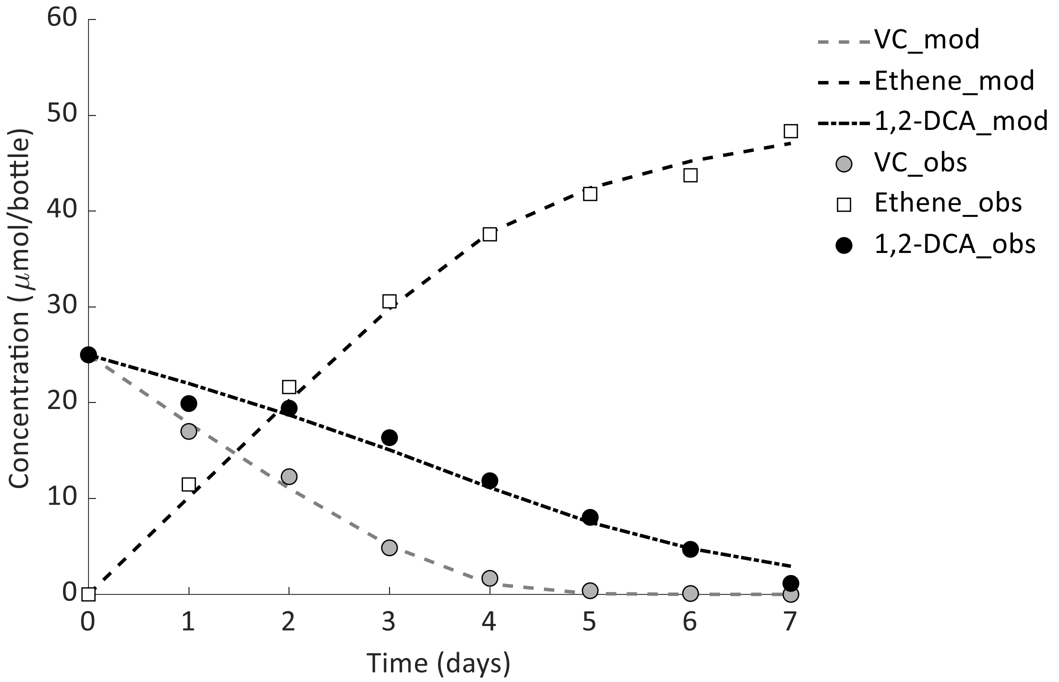

Fig. S9: Modeled (mod) and observed (obs) concentrations in culture EA-T4_A.

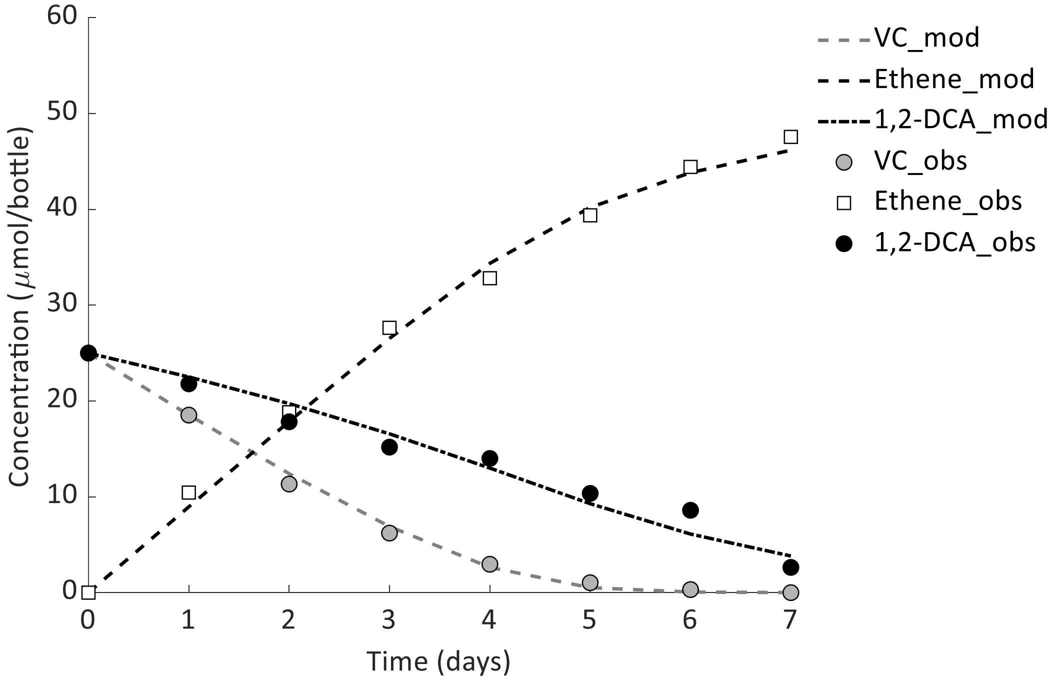

Fig. S10: Modeled (mod) and observed (obs) concentrations in culture EA-T4_B.

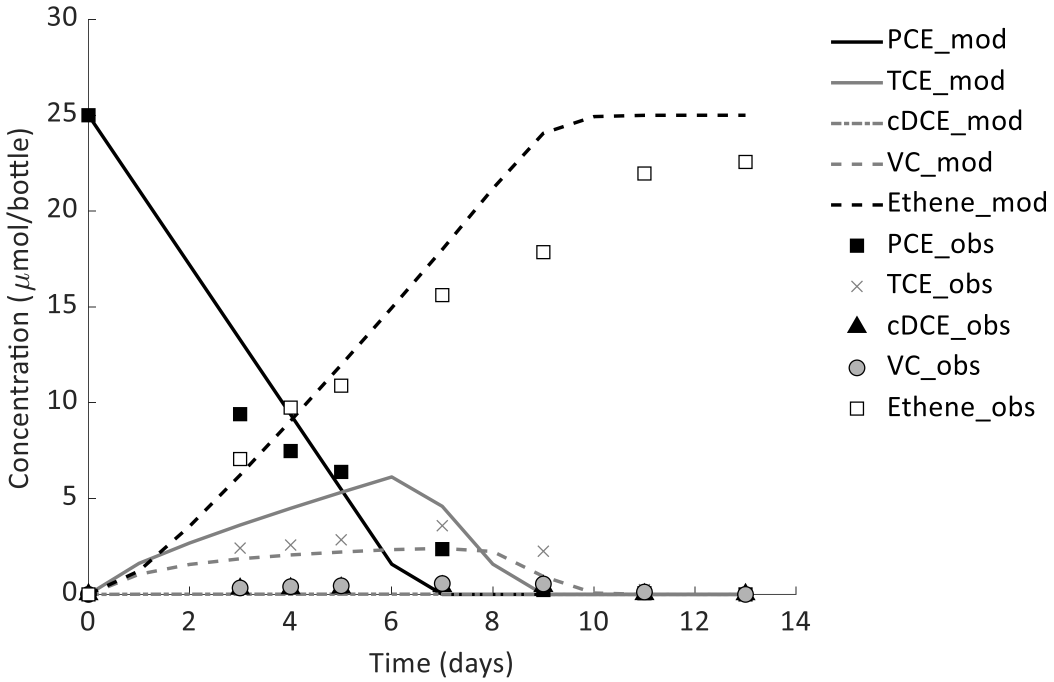

Fig. S11: Modeled (mod) and observed (obs) concentrations in culture EAT5_A.

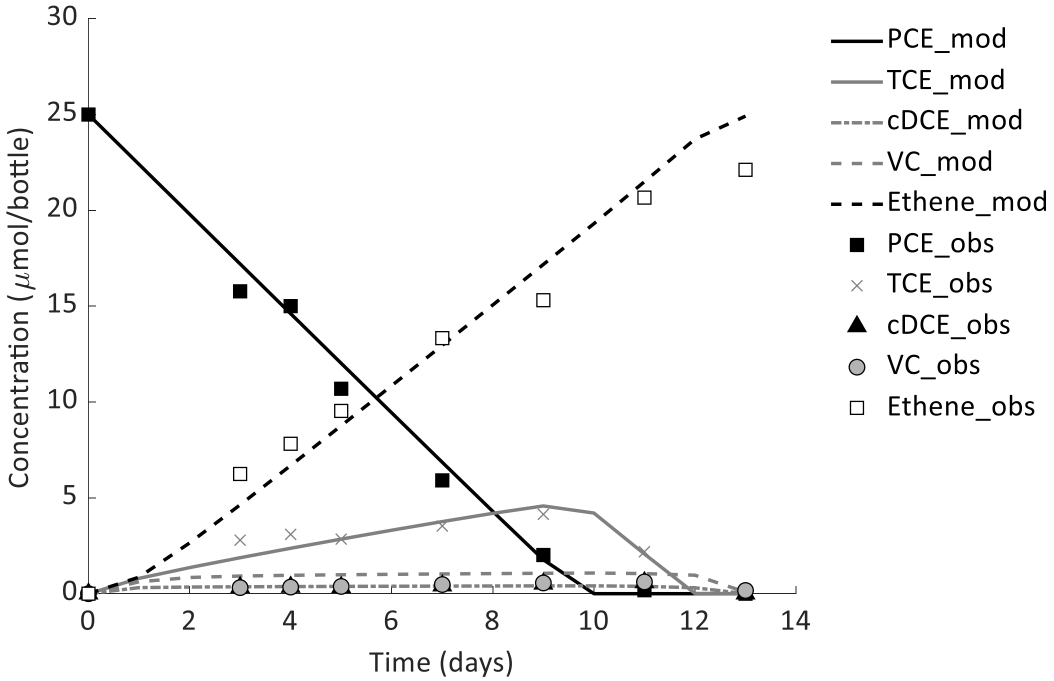

Fig. S12: Modeled (mod) and observed (obs) concentrations in culture EAT5_B.

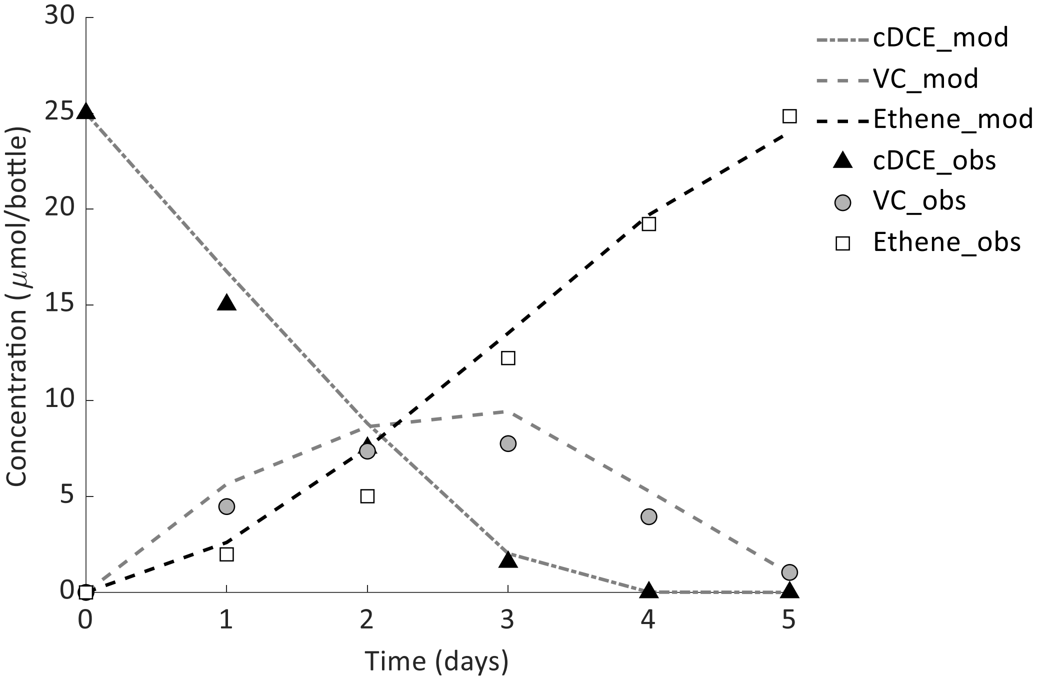

Fig. S13 Modeled (mod) and observed (obs) concentrations in culture EA-T6_A.

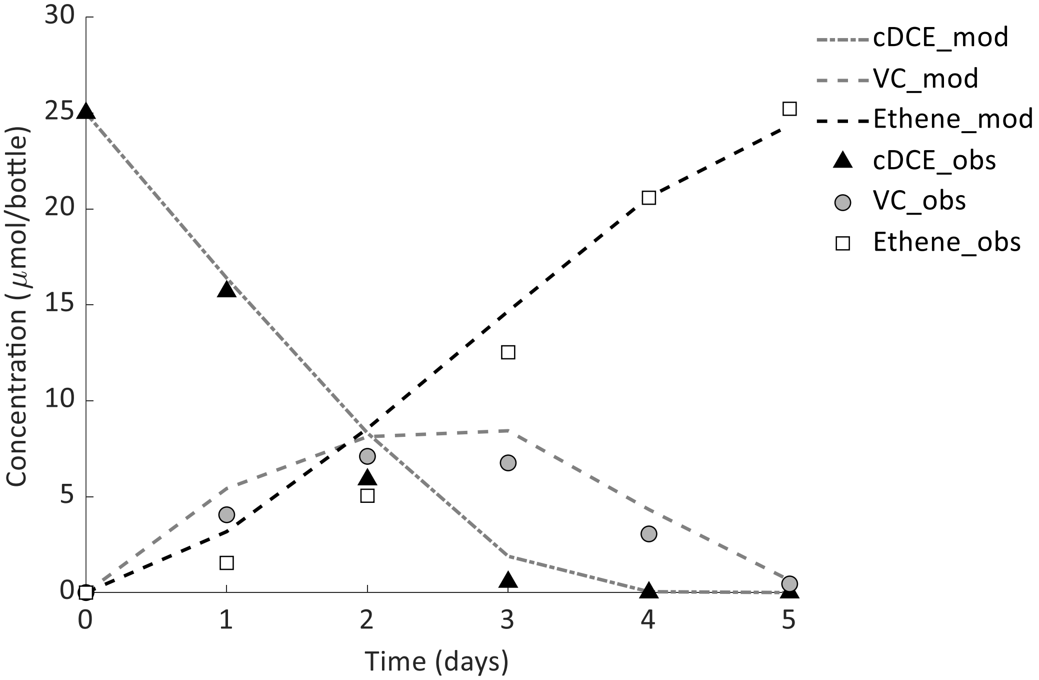

Fig. S14: Modeled (mod) and observed (obs) concentrations in culture EA-T6_B.

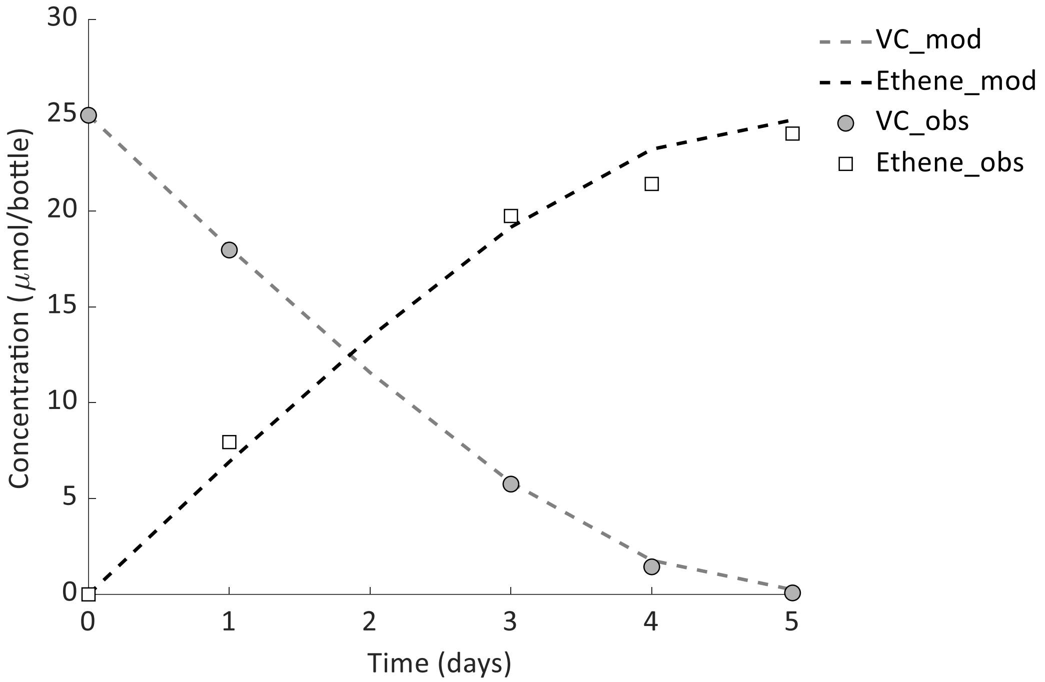

Fig. S15: Modeled (mod) and observed (obs) concentrations in culture EA-T7_A.

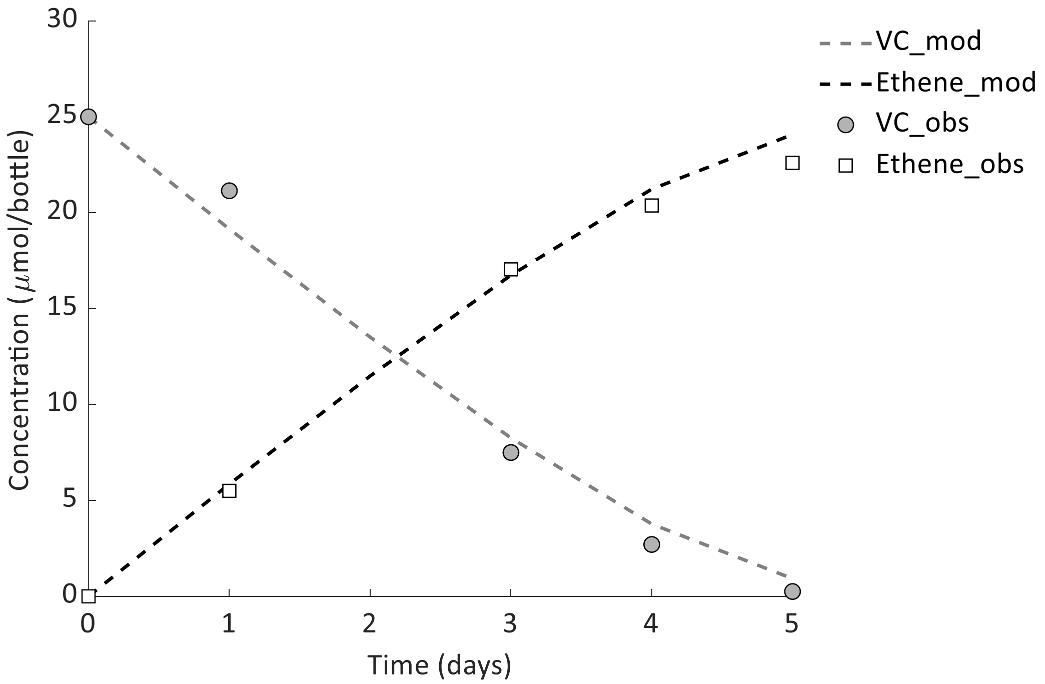

Fig. S16: Modeled (mod) and observed (obs) concentrations in culture EA-T7_B.

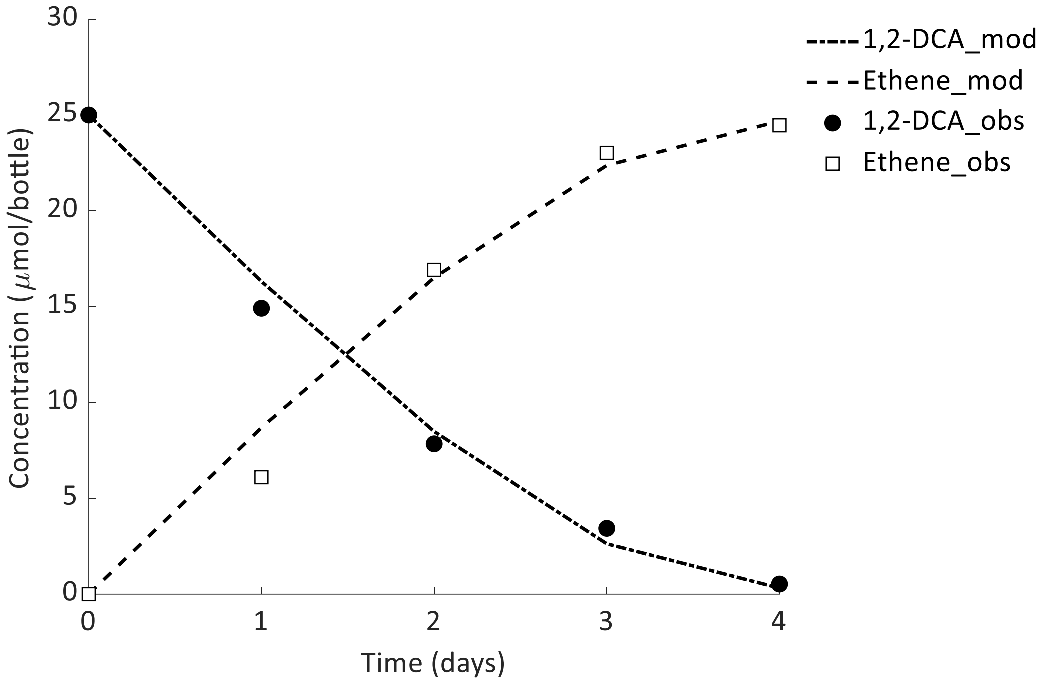

Fig. S17: Modeled (mod) and observed (obs) concentrations in culture EA-T8_A.

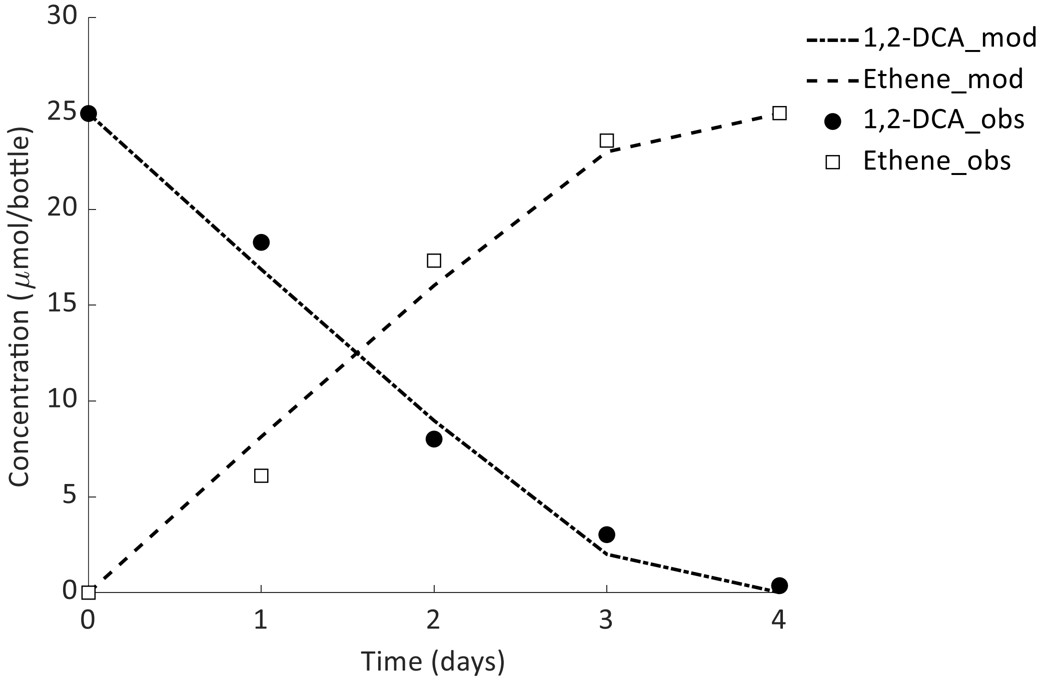

Fig. S18: Modeled (mod) and observed (obs) concentrations in culture EA-T8_B.

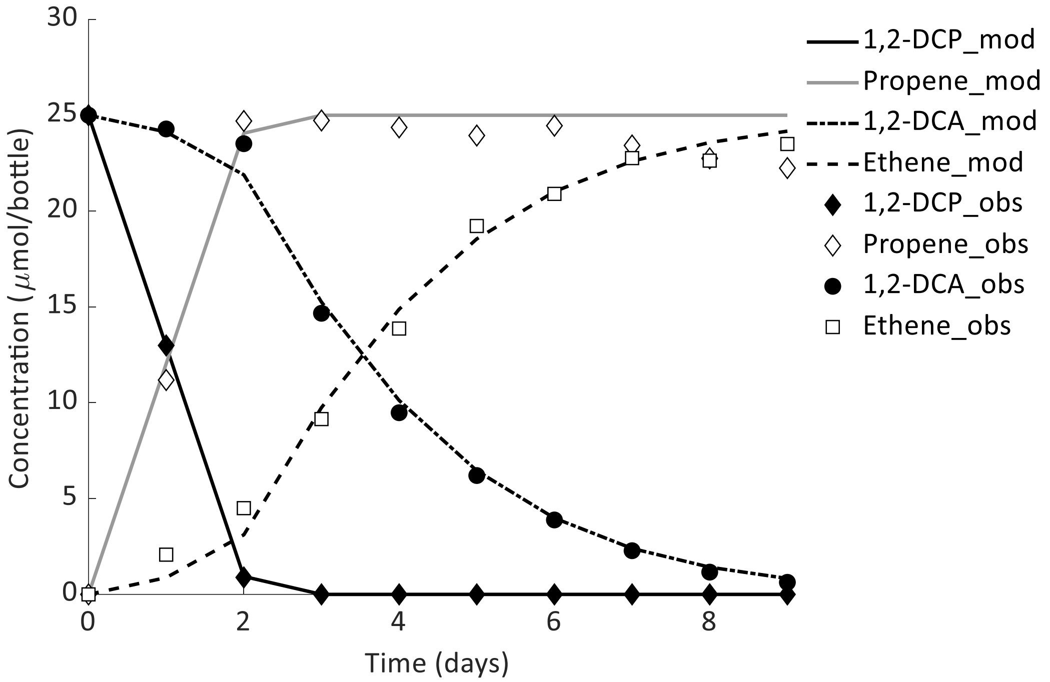

Fig. S19: Modeled (mod) and observed (obs) concentrations in culture EA-T9_A.

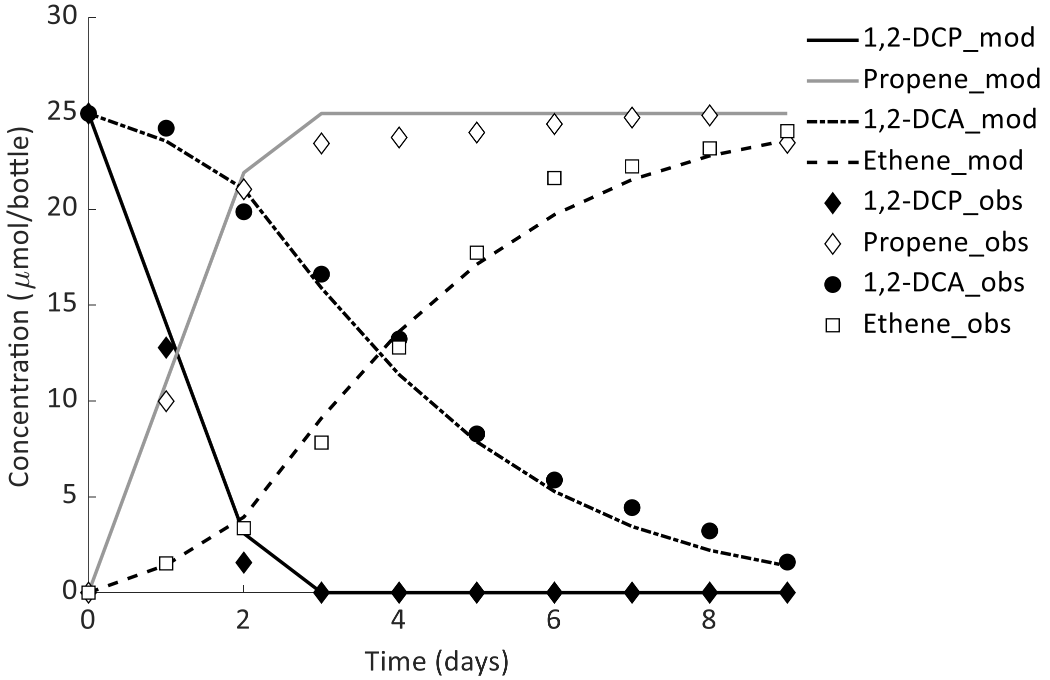

Fig. S20: Modeled (mod) and observed (obs) concentrations in culture EA-T9_B.

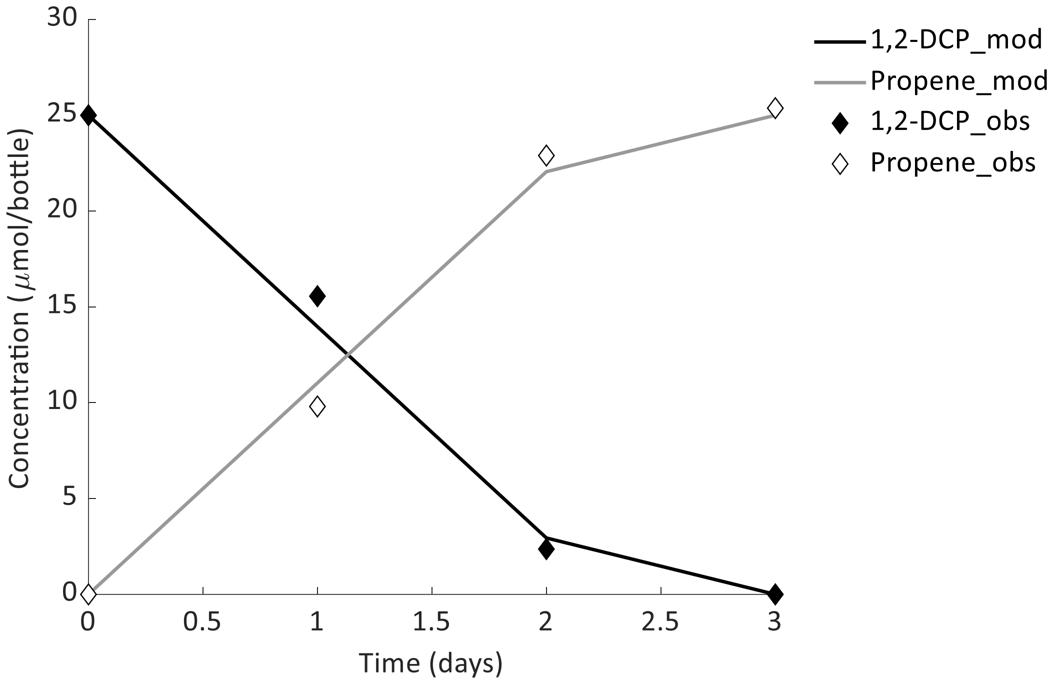

Fig. S21: Modeled (mod) and observed (obs) concentrations in culture EA-T10_A.

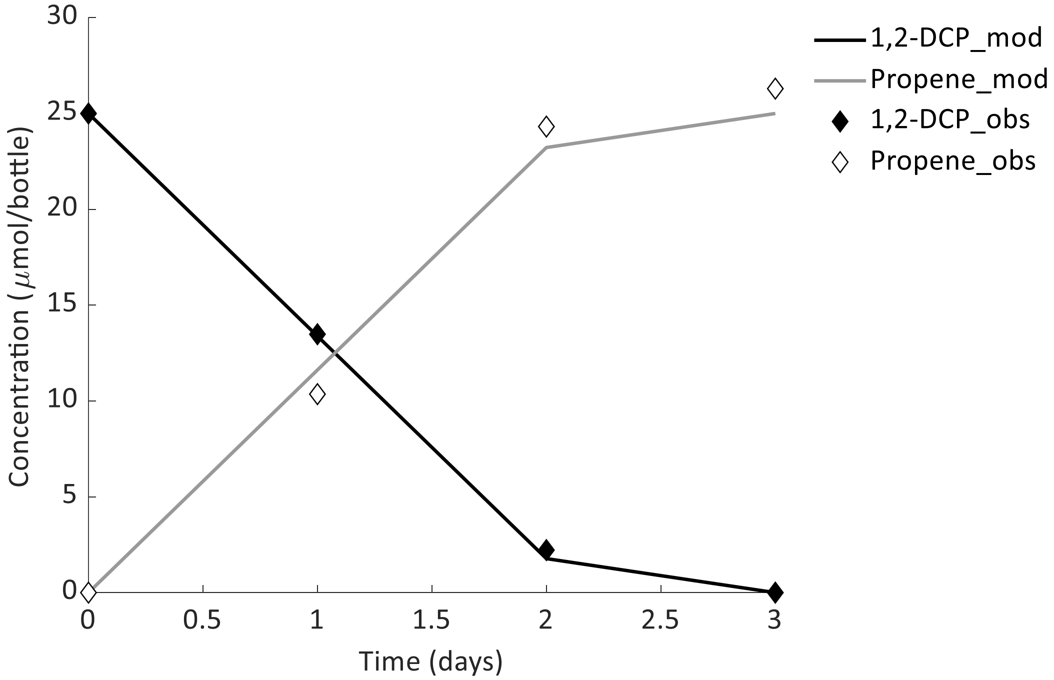

Fig. S22: Modeled (mod) and observed (obs) concentrations in culture EA-T10_B.

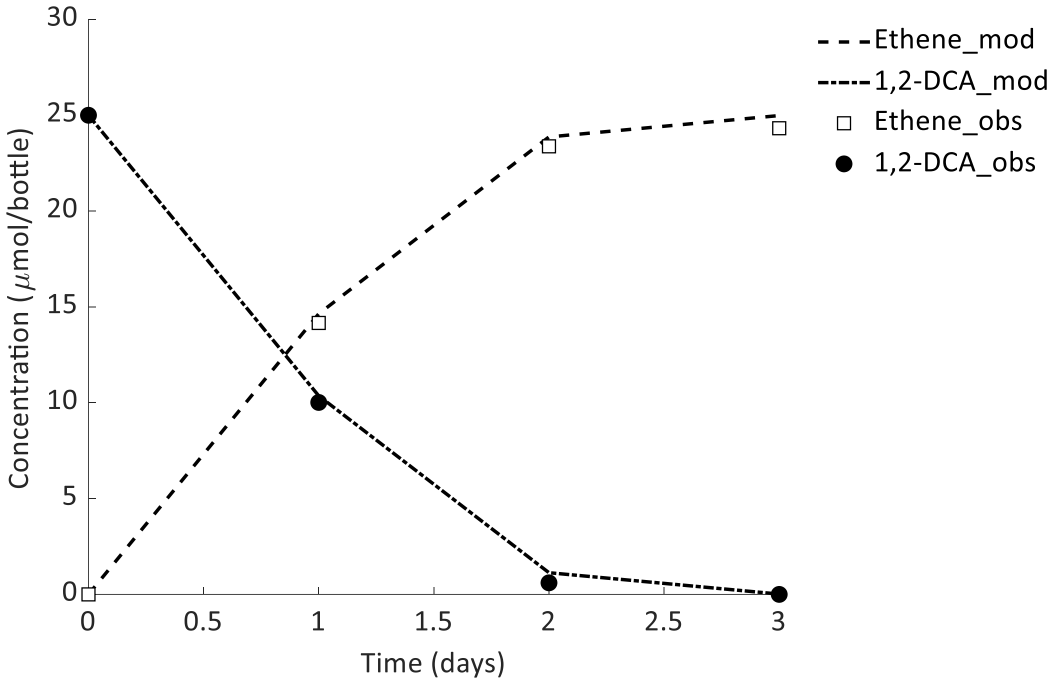

Fig. S23: Modeled (mod) and observed (obs) concentrations in culture EB-T1_A.

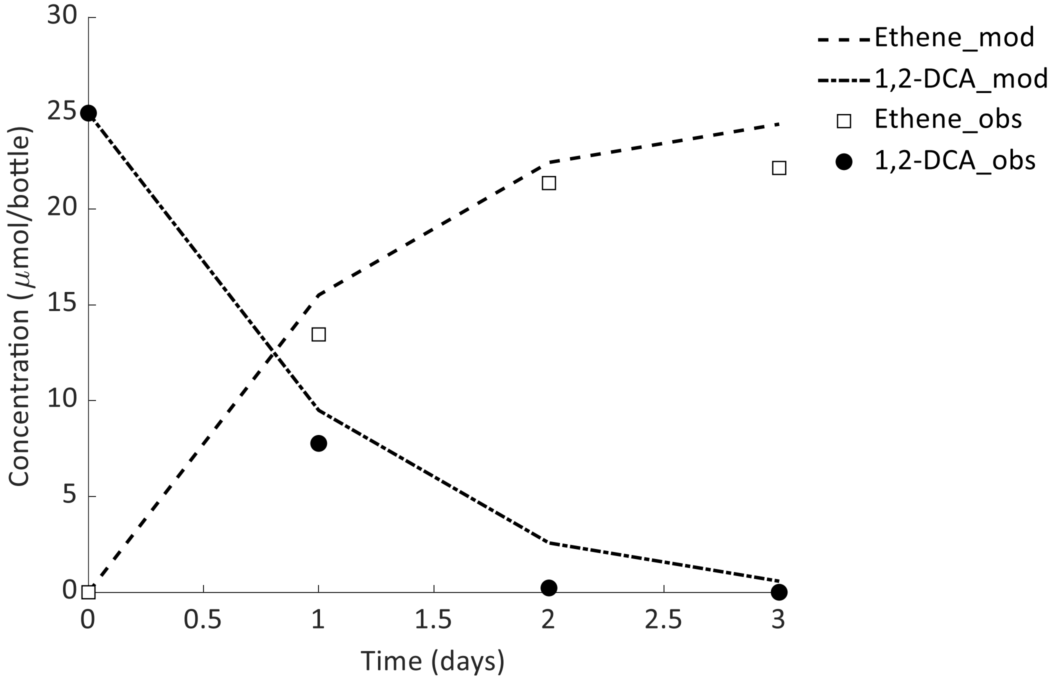

Fig. S24: Modeled (mod) and observed (obs) concentrations in culture EB-T1_B.

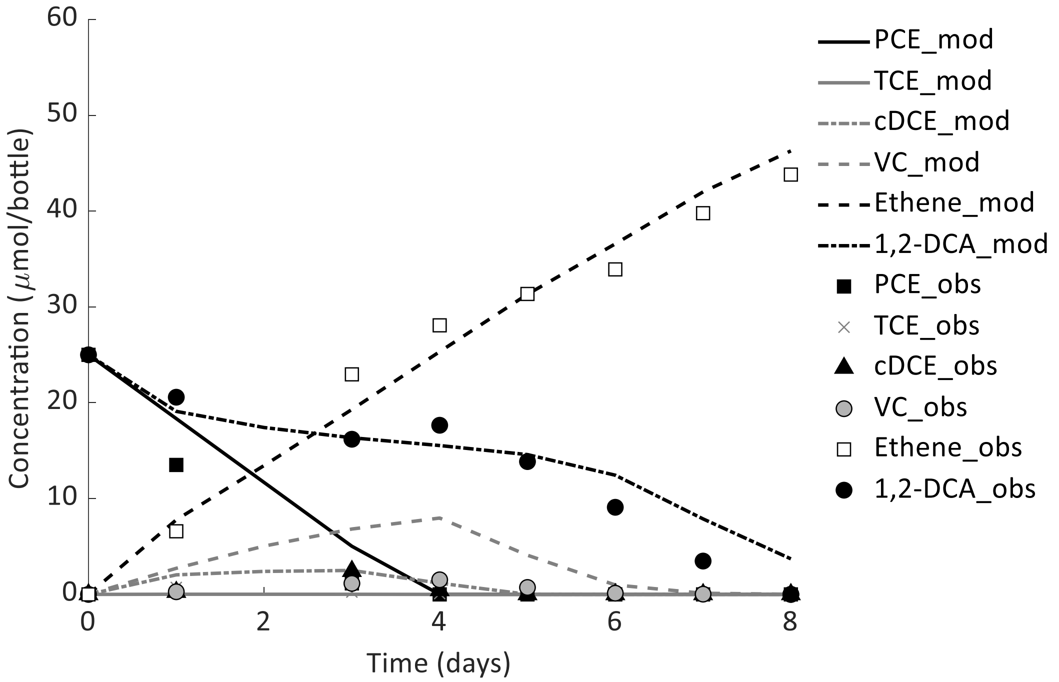

Fig. S25: Modeled (mod) and observed (obs) concentrations in culture EB-T2_A.

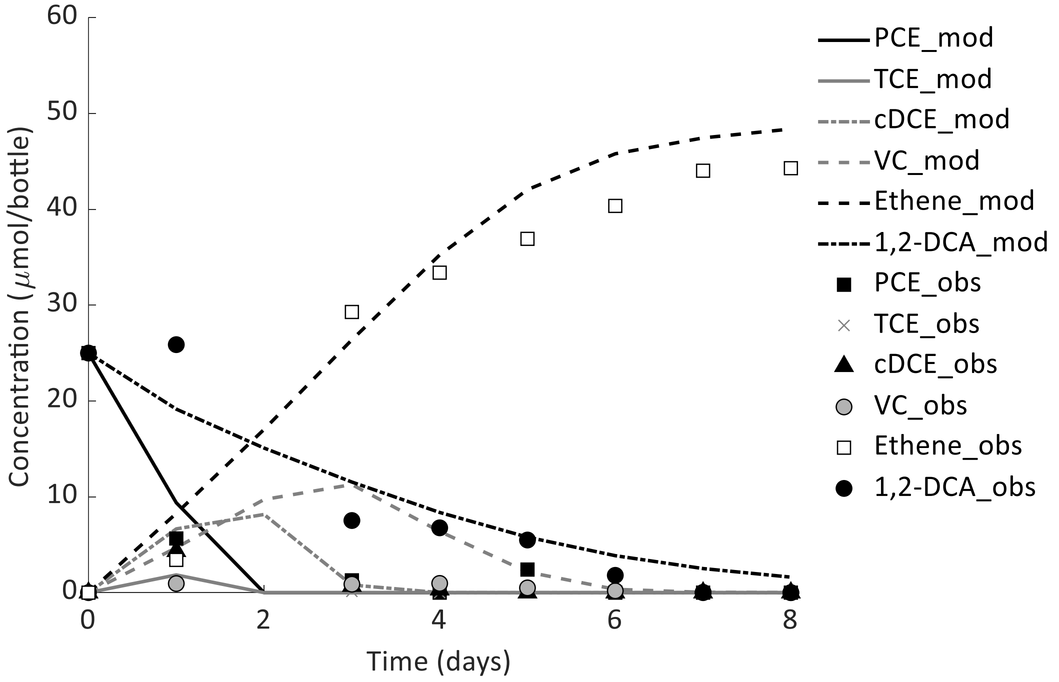

Fig. S26: Modeled (mod) and observed (obs) concentrations in culture EB-T2_B.

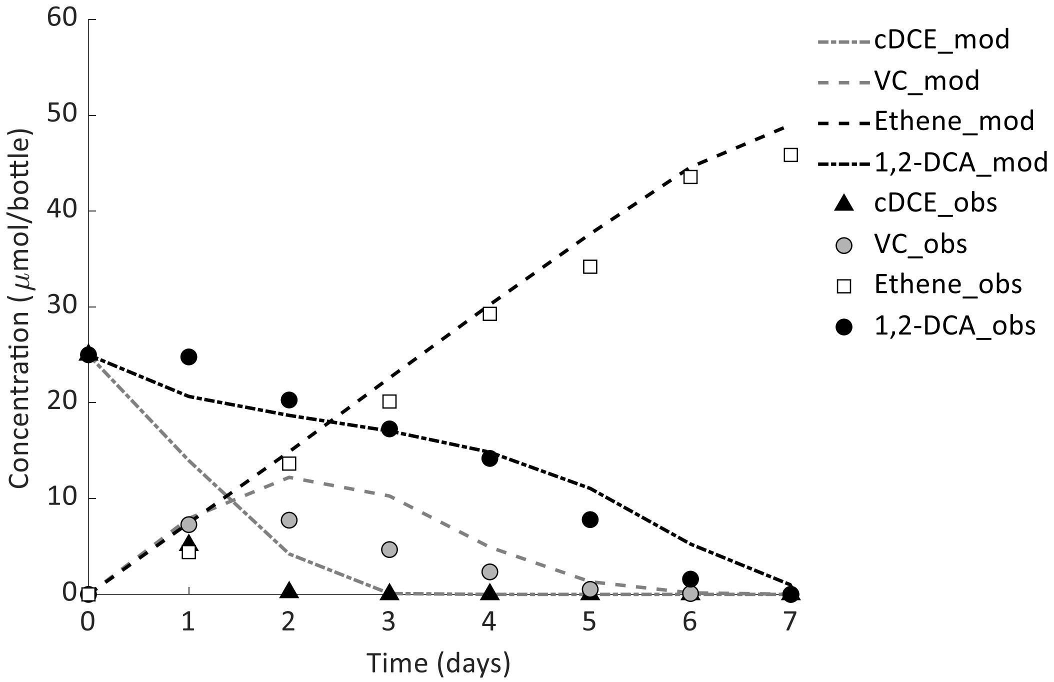

Fig. S27: Modeled (mod) and observed (obs) concentrations in culture EB-T3_A.

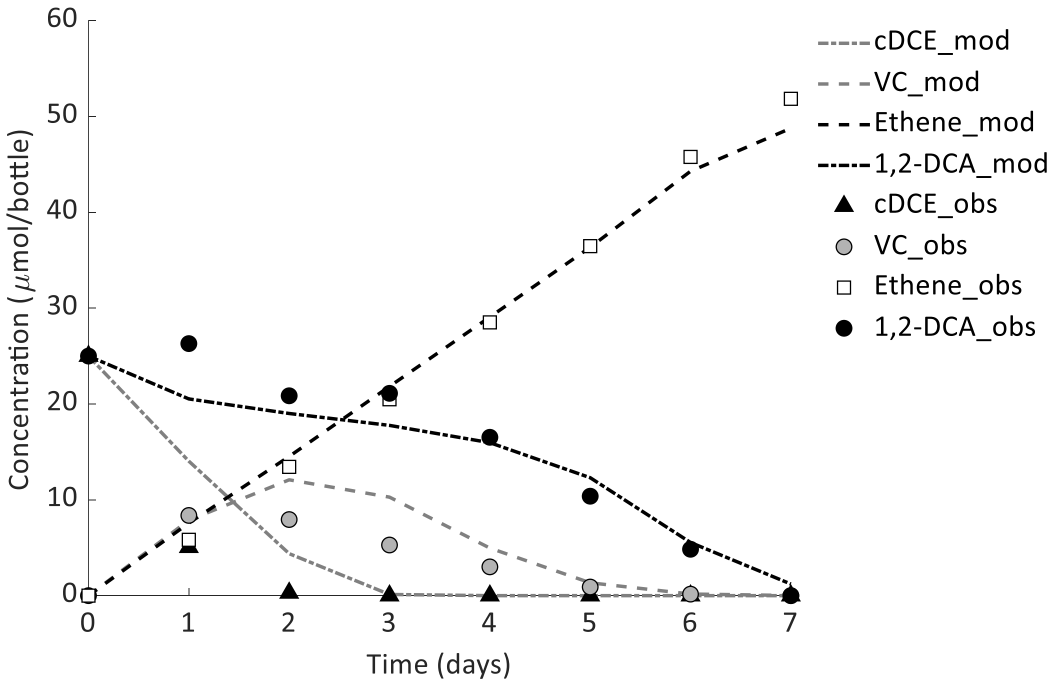

Fig. S28: Modeled (mod) and observed (obs) concentrations in culture EB-T3_B.

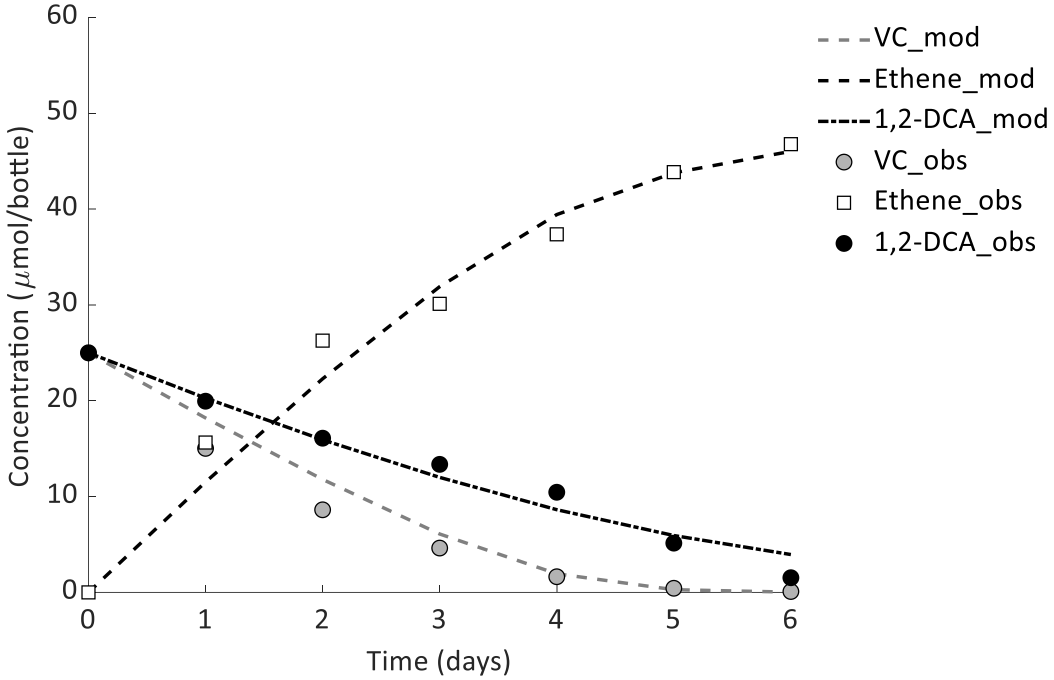

Fig. S29: Modeled (mod) and observed (obs) concentrations in culture EB-T4_A.

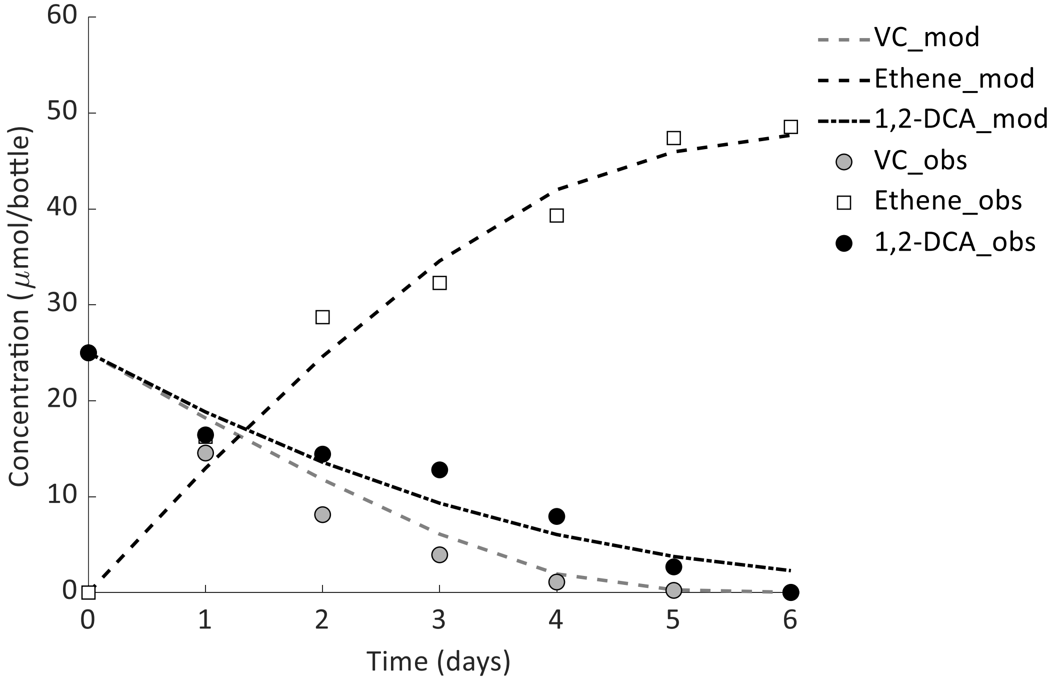

Fig. S30: Modeled (mod) and observed (obs) concentrations in culture EB-T4_B.

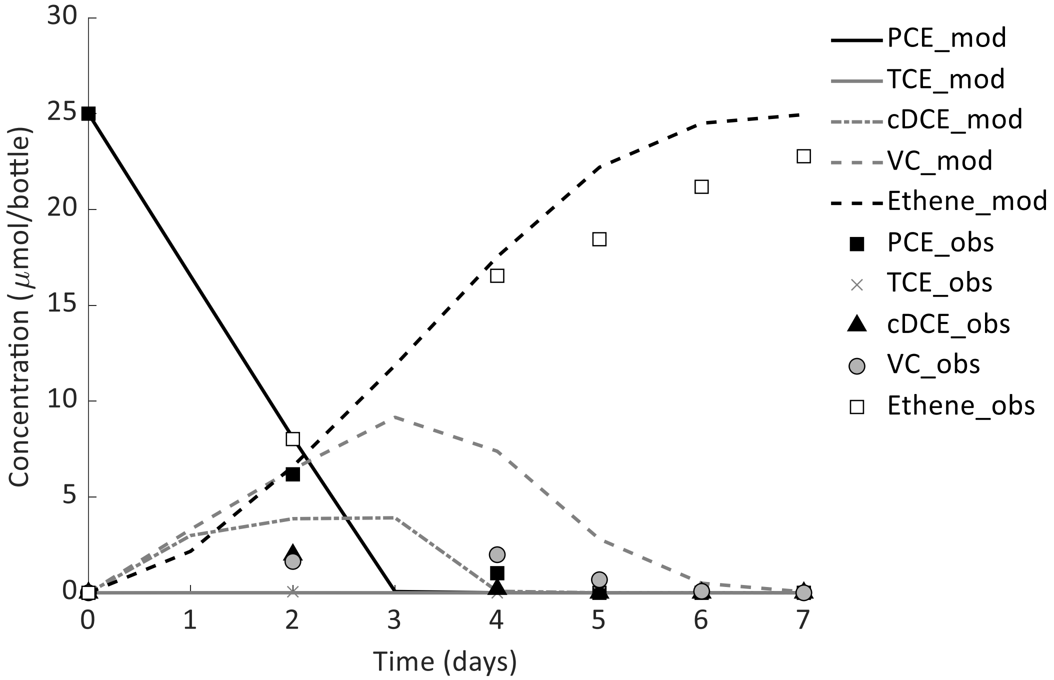

Fig. S31: Modeled (mod) and observed (obs) concentrations in culture EB-T5_A.

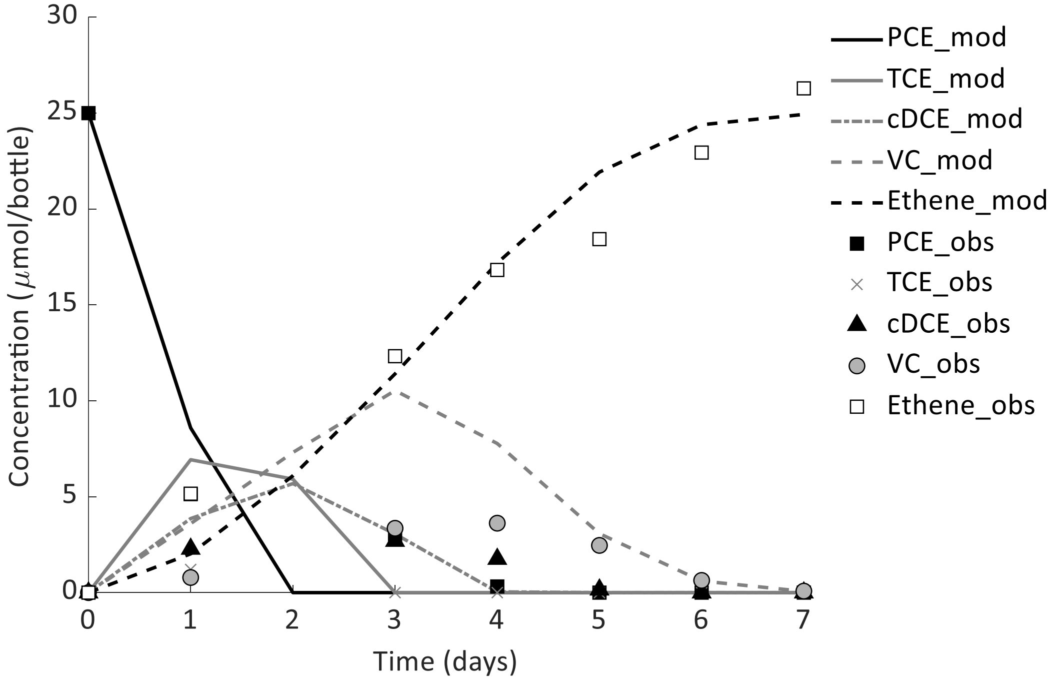

Fig. S32: Modeled (mod) and observed (obs) concentrations in culture EB-T5_B.

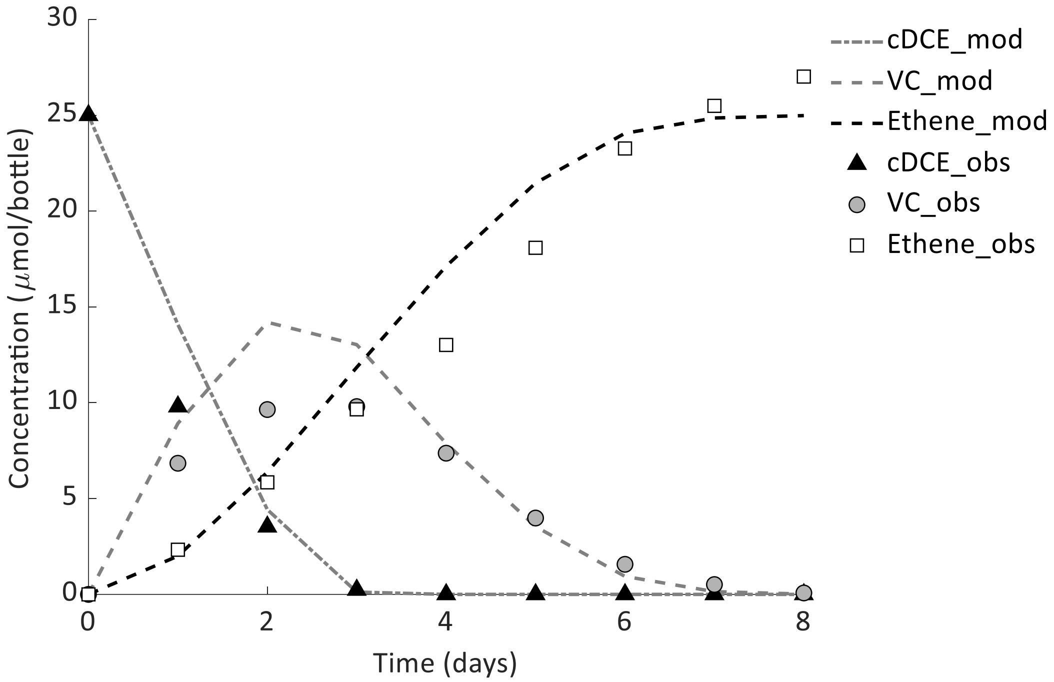

Fig. S33: Modeled (mod) and observed (obs) concentrations in culture EB-T6_A.

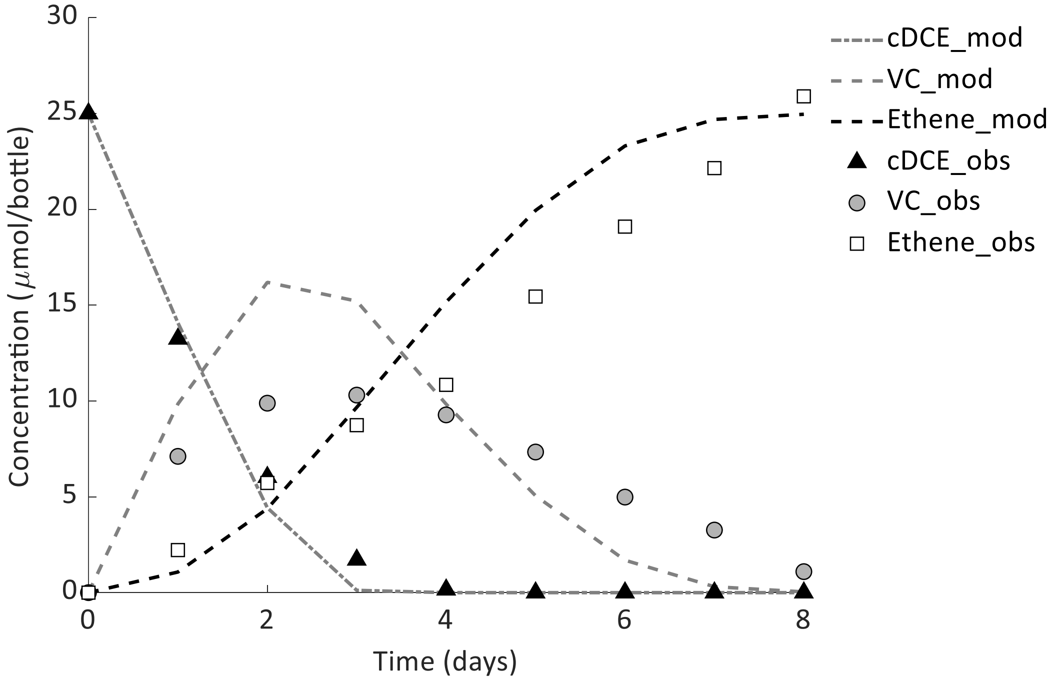

Fig. S34: Modeled (mod) and observed (obs) concentrations in culture EB-T6_B.

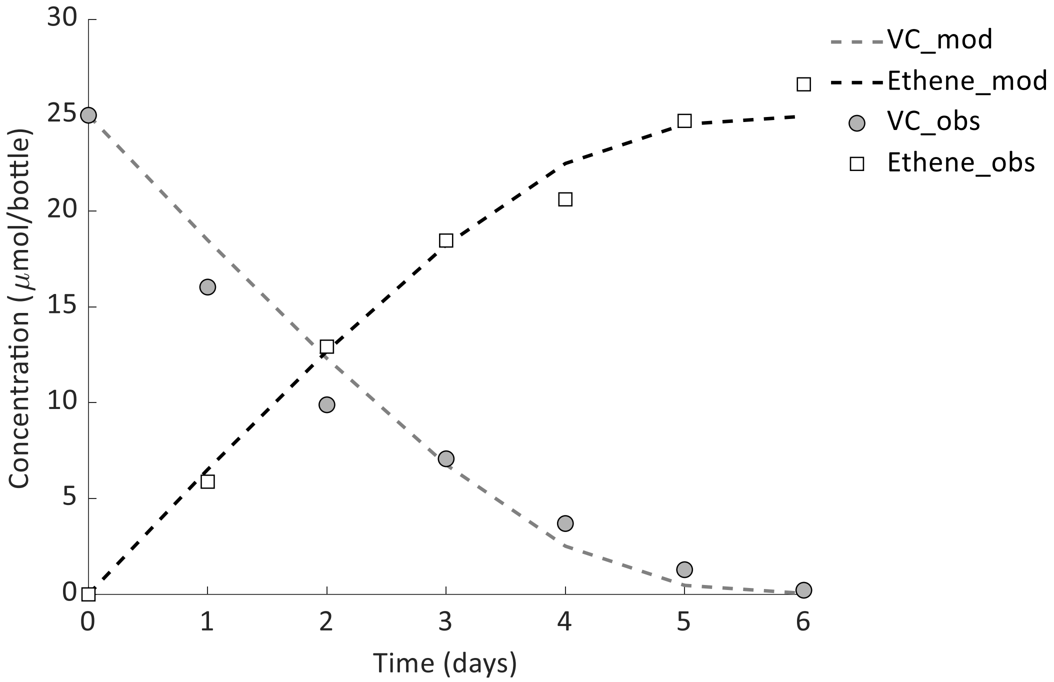

Fig. S35: Modeled (mod) and observed (obs) concentrations in culture EB-T7_A.

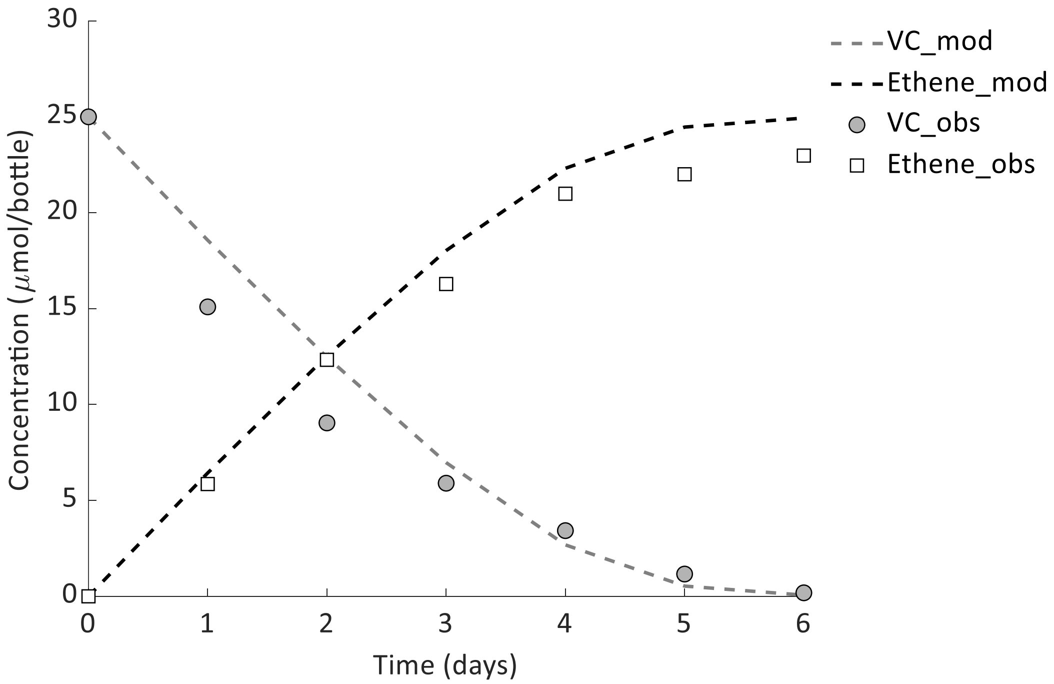

Fig. S36: Modeled (mod) and observed (obs) concentrations in culture EB-T7_B.

Table S1: Primers and amplification programs used for qPCR in this study

Target	Name [a]	Oligonucleotide sequence (5´– 3´)	Reference for primer	Reference for qPCR program
Total bacteria	Eub341F	CCTACGGGAGGCAGCAG	(Muyzer et al. 1993)	(Atashgahi et al. 2013)
	Eub534R	ATTACCGCGGCTGCTGGC		
Dehalogenimonas	BL-DC-1243F	GGYACAATGGGTTGCCACCGG	(Chen et al. 2014)	(Chen et al. 2014) [b]
	BL-DC-1351R	AACGCGCTATGCTGACACGCGT		
Desulfitobacterium	Dsb406F	GTACGACGAAGGCCTTCGGGT	(Smits et al. 2004)	(Smits et al. 2004)
	Dsb619R	CCCAGGGTTGAGCCCTAGGT		
Dehalococcoides	Dco728F	AAGGCGGTTTTCTAGGTTGTCAC	(Smits et al. 2004)	(Atashgahi et al. 2013)
	Dco944R	CTTCATGCATGTCAAAT		
Dehalobacter	Dre441F	GTTAGGGAAGAACGGCATCTGT	(Smits et al. 2004)	(Atashgahi et al. 2013)
	Dre645R	CCTCTCCTGTCCTCAAGCCATA		
Geobacter	Geo196F	GAATATGCTCCTGATTC	(Amos et al. 2007)	(Azizian et al. 2010)
	Geo535R	TAAATCCGAACAACGCTT		
Sulfurospirillum	Sulfuro114F	GCTAACCTGCCCTTTAGTGG	(Sutton et al. 2015)	(Sutton et al. 2015)
	Sulfuro421R	GTTTACACACCGAAATGCGT		

[a] Primer names may not correspond to the original publication

[b] The qPCR program was modified as 98°C for 5 min, followed by 40 cycles of 98°C for 15 s, 68.2°C for 45 s. Melting curves were included from 55°C to 95°C with increments of 0.5°C and 10 s at each step

Table S2 can be found at: https://github.com/mibwurrepo/Peng-et-al-2019

References

Amos BK, Sung Y, Fletcher KE, Gentry TJ, Wu W-M, Criddle CS, Zhou J, Löffler FE (2007) Detection and quantification of *Geobacter lovleyi* strain SZ: implications for bioremediation at tetrachloroethene-and uranium-impacted sites. Appl Environ Microbiol 73(21):6898-6904 doi:10.1128/Aem.01218-07

Atashgahi S, Maphosa F, Doğan E, Smidt H, Springael D, Dejonghe W (2013) Small-scale oxygen distribution determines the vinyl chloride biodegradation pathway in surficial sediments of riverbed hyporheic zones. FEMS Microbiol Ecol 84(1):133-142 doi:10.1111/1574-6941.12044

Azizian MF, Marshall IP, Behrens S, Spormann AM, Semprini L (2010) Comparison of lactate, formate, and propionate as hydrogen donors for the reductive dehalogenation of trichloroethene in a continuous-flow column. J Contam Hydrol 113(1-4):77-92 doi:10.1016/j.jconhyd.2010.02.004

Chen J, Bowman KS, Rainey FA, Moe WM (2014) Reassessment of PCR primers targeting 16S rRNA genes of the organohalide-respiring genus *Dehalogenimonas*. Biodegradation 25(5):747-756 doi:10.1007/s10532-014-9696-z

Muyzer G, De Waal EC, Uitterlinden AG (1993) Profiling of complex microbial populations by denaturing gradient gel electrophoresis analysis of polymerase chain reaction-amplified genes coding for 16S rRNA. Appl Environ Microbiol 59(3):695-700

Smits TH, Devenoges C, Szynalski K, Maillard J, Holliger C (2004) Development of a real-time PCR method for quantification of the three genera *Dehalobacter, Dehalococcoides*, and *Desulfitobacterium* in microbial communities. J Microbiol Methods 57(3):369-378 doi:10.1016/j.mimet.2004.02.003

Sutton NB, Atashgahi S, Saccenti E, Grotenhuis T, Smidt H, Rijnaarts HH (2015) Microbial community response of an organohalide respiring enrichment culture to permanganate oxidation. PLoS One 10(8):e0134615 doi:10.1371/journal.pone.0134615
